# Interaction of Selected Anthracycline and Tetracycline Chemotherapeutics with Poly(I:C) Molecules

**DOI:** 10.1021/acsomega.4c05483

**Published:** 2025-04-15

**Authors:** Markéta Skaličková, Nikita Abramenko, Tatsiana Charnavets, Frédéric Vellieux, Jindřiška Leischner Fialová, Kateřina Kučnirová, Zdeněk Kejík, Michal Masařík, Pavel Martásek, Karel Pacak, Tomáš Pacák, Milan Jakubek

**Affiliations:** † BIOCEV, First Faculty of Medicine, Charles University, 252 50 Vestec, Czech Republic; ‡ Department of Paediatrics and Inherited Metabolic Disorders, First Faculty of Medicine, Charles University and General University Hospital, 120 00 Prague, Czech Republic; § Institute of Biotechnology of the Czech Academy of Sciences, BIOCEV, 252 50 Vestec, Czech Republic; ∥ Department of Physiology, Faculty of Medicine, Masaryk University, Kamenice 5, 625 00 Brno, Czech Republic; ⊥ Department of Pathological Physiology, Faculty of Medicine, Masaryk University, Kamenice 5, Brno CZ-625 00, Czech Republic; # Section on Medical Neuroendocrinology, Eunice Kennedy Shriver National Institute of Child Health and Human Development, National Institutes of Health, Building 10, Room 1-3140, 10 Center Drive, Bethesda, Maryland 20892, United States; ¶ TumorSHOT, Italská 2581/67, Vinohrady, Praha 2, Prague 120 00, Czech Republic

## Abstract

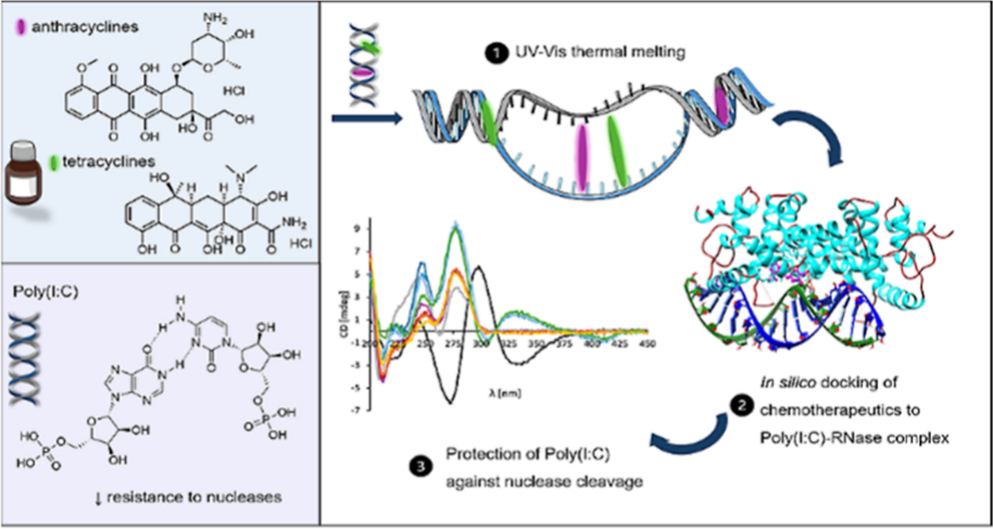

Despite the natural ability of the immune system to recognize cancer and, in some patients, even to eliminate it, cancer cells have acquired numerous evading mechanisms. With the increasing knowledge and focus shifting from targeting rapidly proliferating cells with chemotherapy to modulating the immune system, there have been recent efforts to integrate (e.g., simultaneously or sequentially) various therapeutic approaches. Combining the oncolytic activity of some chemotherapeutics with immunostimulatory molecules, so-called chemoimmunotherapy, is an attractive strategy. An example of such an immunostimulatory molecule is polyinosinic:polycytidylic acid [Poly(I:C)], a synthetic analogue of double-stranded RNA characterized by rapid nuclease degradation hampering its biological activity. This study investigated the possible interactions of tetracycline and anthracycline chemotherapeutics with different commercial Poly(I:C) molecules and protection against nuclease degradation. Fluorescence spectroscopy and circular dichroism revealed an interaction of all of the selected chemotherapeutics with Poly(I:C)s and the ability of doxycycline and minocycline to prolong the resistance to RNase cleavage, respectively. The partial protection was observed *in vitro* as well.

## Introduction

Today, cancer is the second leading cause of death worldwide, with an expected increase of new cases to about 28.4 million in 2040.^1^ Finding efficient drugs with potent anticancer activity, especially in the case of metastatic disease, is a major continuing challenge. Although the immune system can recognize and eliminate cancer cells to some extent, for example, as described in SR/CR mice repeatedly rejecting sarcoma cells,^2,3^ cancer cells have evolved numerous mechanisms to evade the immune system.^4−6^ Therefore, strategies targeting two or more “hallmarks of cancer” or multiple targets in specific cancer pathways are preferable for current treatments to achieve better efficacy.^7^ For instance, combining the oncolytic activity of some chemotherapeutics with immunostimulatory molecules, so-called chemoimmunotherapy, is a compelling therapeutic approach extensively studied and used in preclinical and clinical studies.^8−10^ Combination therapy can also allow lower therapeutic doses, decrease potential side effects, or exert synergy.^11,12^

Polyinosinic:polycytidylic acid, Poly(I:C), is a synthetic analogue of double-stranded RNA (dsRNA) with described immunostimulatory and anticancer activities.^13−16^ Unfortunately, weak homogeneity (e.g., 100–325 kDa for InvivoGen and 151–166 kDa for Sigma-Aldrich)^17^ and mainly rapid serum RNase degradation^18^ hamper the use of commercially available Poly(I:C) molecules in clinical trials. Poly(I:C) stabilized with carboxymethylcellulose and poly l-lysine (so-called Poly-ICLC) that exerts high resistance to serum nucleases and prolonged biological activity^18,19^ and nanoplexed Poly(I:C) (so-called BO-112)^20^ are heavily used in clinical trials. However, they are not commercially available. The reduced resistance of commercial Poly(I:C)s to serum nucleases could be potentially resolved with the simultaneous administration of chemotherapeutics, as proposed for short dsRNAs.^21−23^

Tetracyclines such as doxycycline, minocycline, or tetracycline are antibiotics that interfere with bacterial protein synthesis by binding to 16S rRNA (rRNA) of the 30S prokaryotic ribosomal subunit and prevent the interaction of aminoacyl-tRNA.^24−27^ 16S rRNA naturally folds in the presence of Mg^2+^ ions or ribosomal proteins, so that two rRNA strands interact with each other.^28^ This inspired several studies that reported the interaction of tetracyclines with calf thymus DNA,^29^ short dsRNA,^21^ RNA,^30,31^ or prolonged protection of short dsRNAs against RNase degradation.^21−23^ Besides the antibacterial properties of tetracyclines, anticancer activity in various cancer cell lines, such as breast cancer,^32^ lung cancer,^33^ or melanoma,^34^ has also been described.

In the case of anthracyclines such as doxorubicin hydrochloride, which is approved by the Food and Drug Administration for the treatment of various cancers, the mechanism of anticancer activity is very complex and is suggested to be mediated via DNA intercalation,^35,36^ formation of adducts between DNA strands via hydrogen or covalent bonds, interaction with topoisomerase II–DNA complex, and inhibition of the enzyme activity.^35^ The interaction of anthracyclines with mRNA or DNA was previously described as well.^36−38^

In this study, we combined the selected tetracycline and anthracycline chemotherapeutics with commercially available Poly(I:C) molecules to study their possible interactions and protection of Poly(I:C)s against nuclease degradation.

## Results and Discussion

**Figure 1:**
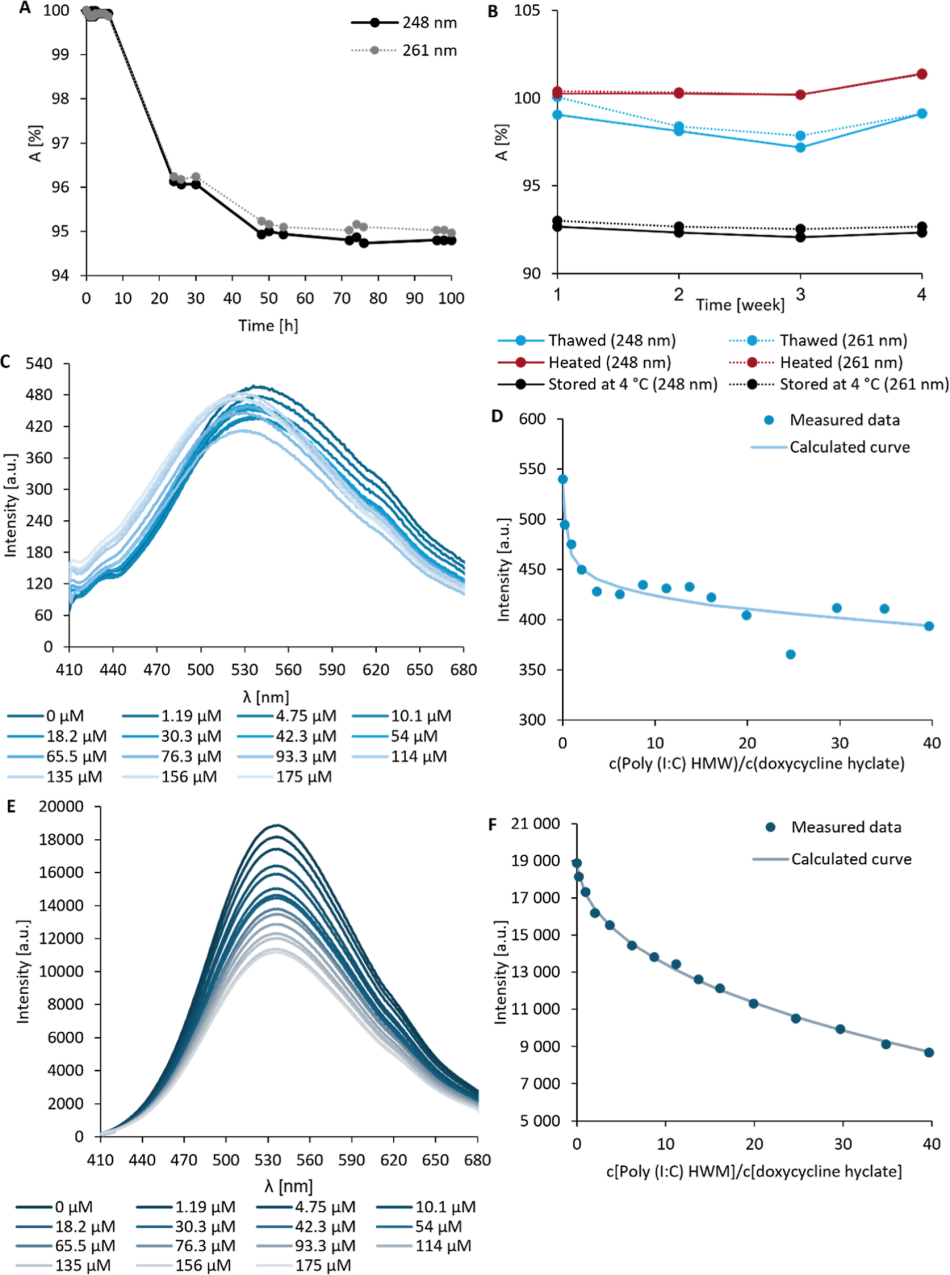
(A) Absorbance changes (%) of Poly(I:C) HMW (100 μg/mL) at 248 and 261 nm maxima for up to 100 h at 4 °C in 0.9% NaCl (pH 5.8) (compared to absorbance at time 0 h (*A*_0h_)). (B) Absorbance changes (%) of Poly(I:C) HMW at the absorbance maxima after storage at 4 °C, repeated freezing (-20 °C), and thawing (50 °C) for 1 month (compared to *A*_0h_ stored at 4 °C in 0.9% NaCl, pH 5.8). (C) Fluorescence emission spectrum and (D) fluorescence curve of doxycycline hyclate at 540 nm after increasing amounts of Poly(I:C) HMW in PBS. (E) Fluorescence emission spectrum and (F) fluorescence curve of doxycycline hyclate at 537 nm after increasing amounts of Poly(I:C) HMW in the presence of 10 mM MgSO_4_ in PBS. The concentration of each chemotherapeutic agent was held constant at 5 × 10^–6^ M. The molar concentration of Poly(I:C) varied in the range from 1.2 × 10^–6^ to 1.75 × 10^–4^ M.

Before any experiments were conducted, the stability of stock solutions of Poly(I:C)s in the recommended 0.9% NaCl solution was assessed. The selected dsRNA analogues were the sodium salt of Poly(I:C) [Poly(I:C) Na^+^], sodium gamma-irradiated salt of Poly(I:C) [Poly(I:C) Na^+^ γ], and high-molecular-weight Poly(I:C) [Poly(I:C) HMW]. A decrease in absorbance was observed for all Poly(I:C)s stored at 4 °C during the period of 1 month. For Poly(I:C) Na^+^ (Figure S1A) and Poly(I:C) HMW (Figure 1A), the absorbance maxima decreased by about 7% compared to the absorbance at time 0h. In the case of Poly(I:C) Na^+^ γ (Figure S1C), the absorbance maxima at 249 and 264 nm dropped about 12% and 8% after 1 month, respectively. Spectral changes were also detected in PBS over 1 week at 4 °C, except for Poly(I:C) HMW (Figure S1E). These results are in line with the recommended storage conditions: -20 °C for Poly(I:C) Na^+^ and Poly(I:C) Na^+^ γ (stability ∼3 years) and 4 and -20 °C for short-time (stability ∼1 month) and long-time (stability ∼1 year) storage of Poly(I:C) HMW, respectively. Furthermore, repeated freeze–thaw cycles over 4 weeks affected the absorbance spectra for Poly(I:C) Na^+^ and Poly(I:C) Na^+^ γ but not for Poly(I:C) HMW. Therefore, freeze–thaw cycles for Poly(I:C) Na^+^ and Poly(I:C) Na^+^ γ should be avoided. We also advise considering the lowest number of cycles when working with Poly(I:C) HMW (maximum of 4).

The presence of a planar naphthacene ring in tetracyclines (intercalation),^29^ along with a planar anthraquinone ring (intercalation), and daunosamine (groove binding) in anthracyclines^39^ implies possible interactions with commercial Poly(I:C) molecules. To study the interaction of anthracycline (e.g., doxorubicin hydrochloride) and tetracycline chemotherapeutics (e.g., tetracycline hydrochloride, doxycycline hyclate, and minocycline hydrochloride) with selected Poly(I:C)s, changes in the intensity of the fluorescence emission spectra of chemotherapeutics after increasing concentrations of Poly(I:C)s were determined in PBS. Spectra were also measured in the presence of 10 mM MgSO_4_ as Mg^2+^ ions have been reported to mediate the drug–nucleic acid interactions.^21,40,41^ The intensity of the fluorescence emission spectra of chemotherapeutics was found to decrease upon the addition of increasing concentrations of Poly(I:C) Na^+^ (Figures S2A,B, S3A,B, and S5A,B), Poly(I:C) Na^+^ γ (Figure S3C,D), and Poly(I:C) HMW (Figures 1C–D and S3E,F, S5E,F), except for the addition of Poly(I:C) Na^+^ γ to tetracycline (Figure S5C,D) and doxycycline (Figure S2C,D). Such a decrease in fluorescence intensity indicates groove binding or electrostatic interaction (i.e., interaction with the sugar–phosphate backbone).^42^ Contrarily, the increasing fluorescence intensity of tetracycline (Figure S5C,D) and doxycycline (Figure S2C,D) upon the rising concentrations of Poly(I:C) Na^+^ γ suggest the protection of drug molecules from the polar solvent and the intercalation type of interaction.^42^ Furthermore, a hypsochromic (blue) shift was observed for doxycycline and tetracycline after the addition of all Poly(I:C)s and indicates their intercalation between dsRNA bases.^43,44^ Therefore, these data suggest that tetracyclines may interact with Poly(I:C) Na^+^ γ through intercalation, and with Poly(I:C) Na^+^ and Poly(I:C) HMW via both intercalation and groove binding or electrostatic interactions. These results are in agreement with that of Khan and Musarrat.^29^ The addition of MgSO_4_ further increased the fluorescence of chemotherapeutics (Figures 1E,F and S2E–H, S6A–F), except for doxorubicin (Figure S4A–F). Such observation suggests that Mg^2+^ ions may enhance the interactions between Poly(I:C)s and chemotherapeutics.^21,40^ Minocycline hyclate exerted a weak fluorescence signal in PBS. Therefore, the binding constant values of the minocycline–dsRNA complex were not determined.

To determine the strength of interaction between chemotherapeutic compounds and dsRNA analogues in the presence and absence of 10 mM MgSO_4_, we calculated the binding constant values (Table 1) from the measured fluorescence emission spectra.^45^ For most of the chemotherapeutics, the lowest binding affinity was found for Poly(I:C) Na^+^ γ in the absence of Mg^2+^ ions. We suggest that weaker interactions of chemotherapeutics with Poly(I:C) Na^+^ γ may be influenced by the stability of dsRNA strands.^29^ In the case of Poly(I:C) Na^+^ γ, Merck, a minimal irradiation dose of 2.5 Mrad (25KGy) was applied for sterilization.^46^ Radiation causes single- and double-strand breaks^47^ and influences the melting temperature.^48^ The higher the irradiation dose, the lower the melting temperature.^48^ Unfortunately, the highest radiation dose is not further specified for the product. Supplementation with MgSO_4_ led to an increase in the binding affinity of all tested chemotherapeutics to selected Poly(I:C)s, except for the interaction of doxycycline hyclate with Poly(I:C) HMW.

**Table 1:** Binding Constants and Complex Stoichiometry of Selected Chemotherapeutics with dsRNA Analogues in the Presence and Absence of 10 mM MgSO_4_

	with 0 mM MgSO_4_			with 10 mM MgSO_4_	
	Log(K)	stoichiometry (Poly(I:C):chemotherapy)		Log(K)	stoichiometry (Poly(I:C):chemotherapy)
**Poly(I:C) Na**^**+**^			**Poly(I:C) Na**^**+**^		
doxorubicin HCl	10.69 ± 1.14 (595 nm)	1:2	doxorubicin HCl	11.00 ± 1.45 (595 nm)	1:2
	5.03 ± 0.75 (595 nm)	1:1		4.78 ± 1.04 (595 nm)	1:1
doxycycline hyclate	9.85 ± 0.99 (540 nm)	1:2	doxycycline hyclate	12.08 ± 1.37 (537 nm)	1:2
	0.20 ± 0.0041 (540 nm)	1:1		5.39 ± 0.96 (537 nm)	1:1
tetracycline HCl	11.18 ± 0.99 (549 nm)	1:2	tetracycline HCl	11.67 ± 1.47 (528 nm)	1:2
	4.64 ± 0.96 (549 nm)	1:1		5.00 ± 1.16 (528 nm)	1:1
**Poly(I:C) Na**^**+**^**γ**			**Poly(I:C) Na**^**+**^**γ**		
doxorubicin HCl	10.13 ± 1.23 (595 nm)	1:2	doxorubicin HCl	10.58 ± 0.78 (595 nm)	1:2
	4.50 ± 0.72 (595 nm)	1:1		4.49 ± 0.55 (595 nm)	1:1
doxycycline hyclate	10.39 ± 1.8 (540 nm)	1:2	doxycycline hyclate	11.88 ± 1.53 (537 nm)	1:2
	4.14 ± 0.57 (540 nm)	1:1		5.01 ± 0.0764 (537 nm)	1:1
tetracycline HCl	9.57 ± 0.71 (505 nm)	1:2	tetracycline HCl	13.11 ± 1.48 (528 nm)	1:2
	4.37 ± 0.56 (505 nm)	1:1		5.60 ± 1.15 (528 nm)	1:1
**Poly(I:C) HMW**			**Poly(I:C) HMW**		
doxorubicin HCl	11.23 ± 1.10 (595 nm	1:2	doxorubicin HCl	12.87 ± 1.41 (595 nm)	1:2
	5.21 ± 0.75 (595 nm)	1:1		5.64 ± 0.0253 (595 nm)	1:1
doxycycline hyclate	11.34 ± 1.81 (540 nm	1:2	doxycycline hyclate	11.27 ± 1.15 (537 nm)	1:2
	4.62 ± 0.3 (540 nm)	1:1		4.68 ± 0.79 (537 nm)	1:1
tetracycline HCl	11.70 ± 1.37 (549 nm)	1:2	tetracycline HCl	15.89 ± 0.54 (528 nm)	1:2
	5.02 ± 1.05 (549 nm)	1:1		6.98 ± 0.36 (528 nm)	1:1

Intercalating drugs stabilize DNA molecules and increase their melting temperatures,^49^ in contrast to groove binding, which causes slight or no changes in melting temperatures.^50,51^ Therefore, to understand the effects of these interactions, the melting temperatures of dsRNA analogues alone and in combination with chemotherapeutics in the presence and absence of MgSO_4_ were determined. As summarized in Table 2, the melting temperatures of Poly(I:C) Na^+^ and Poly(I:C) HMW were 65 and 67 °C, respectively. An increase of 9 °C in both Poly(I:C)s was observed in the presence of 10 mM MgSO_4_. The chemotherapeutics did not affect the melting temperatures in the absence or presence of 10 mM MgSO_4_. The melting temperatures were increased by the presence of Mg^2+^ ions, except for Poly(I:C) Na^+^ in combination with minocycline hydrochloride. Measurements for Poly(I:C) Na^+^ γ could not be determined due to its significant molecular weight variability.

**Table 2:** Melting Temperatures of dsRNA Analogues Alone and in Combination with Chemotherapeutics in the Absence and Presence of 10 mM MgSO_4_ in PBS

combination with	type of Poly(I:C)	melting temperature [°C]	type of Poly(I:C)	melting temperature [°C]
	with 0 mM MgSO_4_		with 10 mM MgSO_4_	
Poly(I:C) alone	Poly(I:C) Na^+^ (267 nm)	65 ± 0.1 °C	Poly(I:C) Na^+^ (267 nm)	74 ± 0.1 °C
	Poly(I:C) HMW (264 nm)	67 ± 0.1 °C	Poly(I:C) HMW (264 nm)	76 ± 0.1 °C
tetracycline HCl	Poly(I:C) Na^+^	64 ± 0.1 °C	Poly(I:C) Na^+^	73 ± 0.1 °C
	Poly(I:C) HMW	67 ± 0.1 °C	Poly(I:C) HMW	74 ± 0.1 °C
doxycycline hyclate	Poly(I:C) Na^+^	64 ± 0.1 °C	Poly(I:C) Na^+^	73 ± 0.1 °C
	Poly(I:C) HMW	67 ± 0.1 °C	Poly(I:C) HMW	75 ± 0.1 °C
doxorubicin HCl	Poly(I:C) Na^+^	65 ± 0.1 °C	Poly(I:C) Na^+^	72 ± 0.1 °C
	Poly(I:C) HMW	66 ± 0.1 °C	Poly(I:C) HMW	74 ± 0.1 °C
minocycline HCl	Poly(I:C) Na^+^	65 ± 0.1 °C	Poly(I:C) Na^+^	66 ± 0.1 °C
	Poly(I:C) HMW	66 ± 0.1 °C	Poly(I:C) HMW	74 ± 0.1 °C

**Figure 2:**
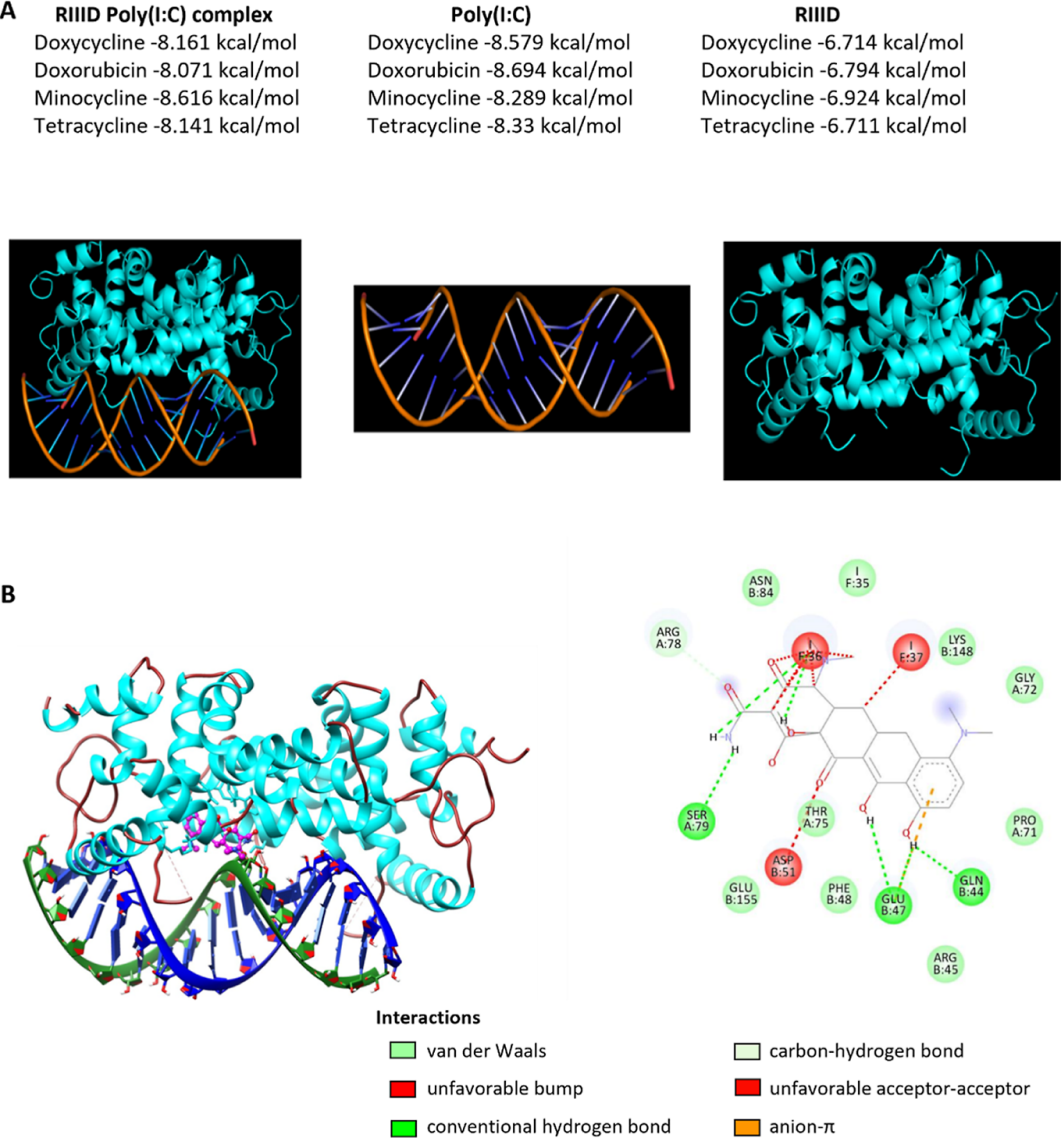
(A) Computed free binding energies between chemotherapy and Poly(I:C) or RIIID and their complex. (B) General view of the docking position of minocycline with the RIIID–Poly(I:C) complex (left) and 2D diagram of interactions (right).

Before studying the ability of the selected chemotherapeutics to protect dsRNA analogues against nuclease degradation, in silico docking was conducted. Molecular docking studies of ligands (i.e., chemotherapeutic agents) were performed separately for the complex of a human nuclease RIIID with double-stranded Poly(I:C), as well as for RIIID and Poly(I:C) alone (Figures 2 and S7). This approach allowed for a detailed comparison of interactions within the complex and between each component. The computed free binding energies between Poly(I:C) and doxorubicin hydrochloride, tetracycline hydrochloride, minocycline hydrochloride, and doxycycline hyclate were -8.694, -8.33, -8.289, and -8.579 kcal/mol, respectively (Figure 2A). A better docking score was achieved for the interaction of chemotherapeutics to the Poly(I:C) molecule (Figure 2A). Therefore, the computed data suggest a possible mode of protection of Poly(I:C) against nuclease degradation mediated via the interaction of chemotherapeutics to the molecule that is about to be cleaved by a nuclease. Figures 2B and S7 show the potential binding sites where the interaction between Poly(I:C) and RIIID could be disrupted by the ligands (i.e., chemotherapeutics). Unfavorable interactions are depicted in red and hinder the interaction of selected chemotherapeutics with the RIIID–Poly(I:C) complex. Beneficial, i.e., favorable, interactions are depicted in bright green color.

**Figure 3:**
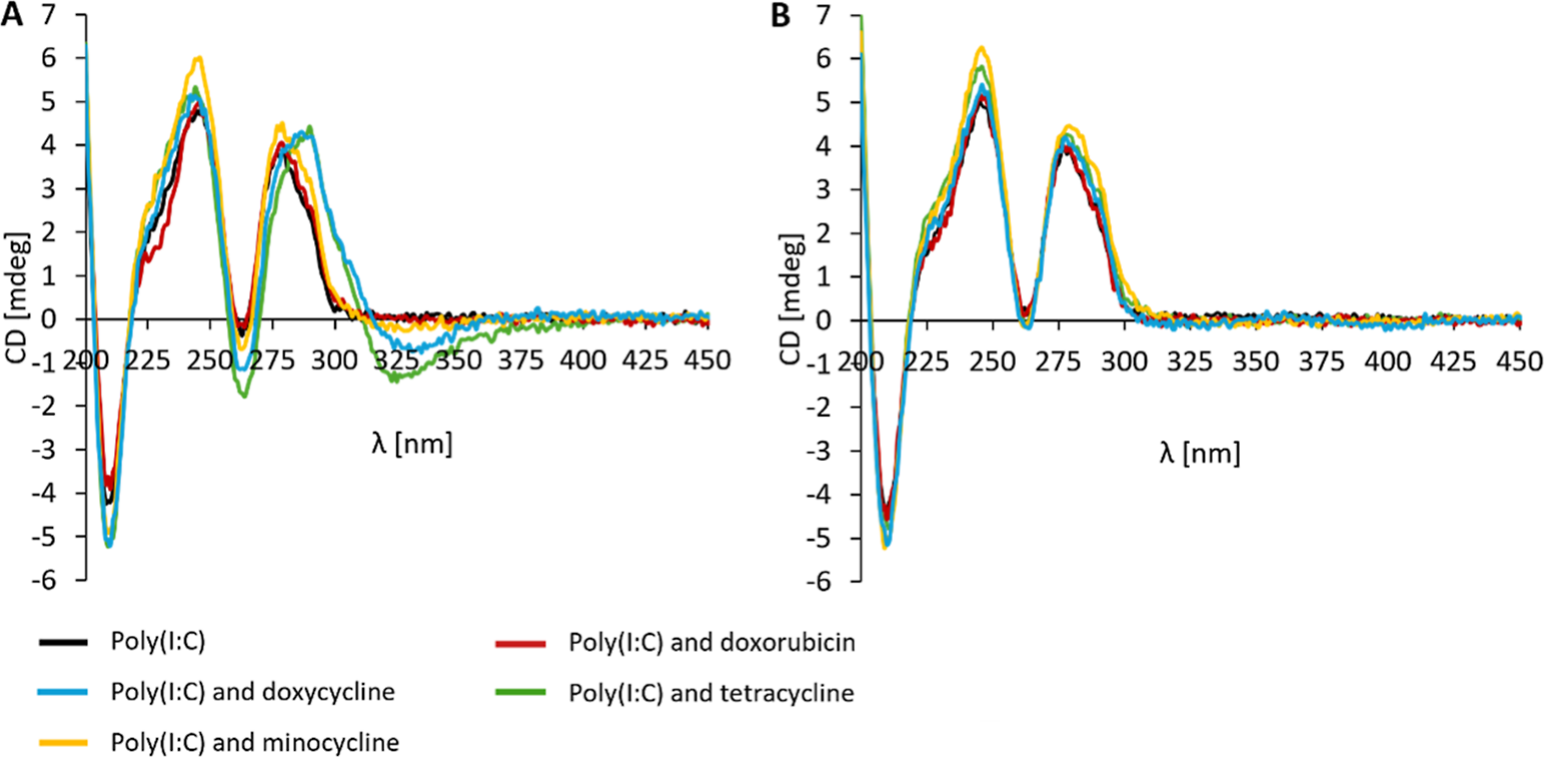
(A) CD spectra of chemotherapeutics, Poly(I:C) HMW, and their combinations in the absence of 10 mM MgSO_4_ (B) and presence of 10 mM MgSO_4_ in PBS. The concentration of Poly(I:C)s and chemotherapeutics was 1 × 10^–4^ M (1:1).

Following the experiments, circular dichroism (CD) was used to study the effects of chemotherapeutic interactions on the secondary structure of dsRNA and the protection against RNase degradation. The CD spectra of all selected Poly(I:C)s exhibited two positive bands around 245 and 280 nm, characteristic of dsRNAs.^21,52,53^ Upon the addition of chemotherapeutics to Poly(I:C)s, spectral changes were detected, including bathochromic (i.e., red) shifts and variations in CD intensity. Specifically, doxycycline and tetracycline induced a red shift to 295 nm in the CD spectra of Poly(I:C) Na^+^ (Figure S9A), Poly(I:C) Na^+^ γ (Figure S9C), and Poly(I:C) HMW (Figure 3A). In the case of minocycline (Figure 3), a slight hyperchromicity of Poly(I:C) HMW around 245 nm was observed. Such spectral changes suggest the interaction of doxycycline, minocycline, and tetracycline with Poly(I:C) and changes in the dsRNA structure. For instance, increased ellipticity was described to be characteristic of duplex elongation and intercalation type of interaction.^54−56^ The addition of MgSO_4_ induced intensity changes in the CD spectra with no spectral shifts. Furthermore, the measured CD spectra were compared to the calculated spectra, i.e., a sum of individual Poly(I:C) and chemotherapy components, reflecting a noninteracting state. Differences in the ellipticity and peak shifts between the measured and calculated CD spectra further suggest Poly(I:C)–chemotherapy binding interactions (Figures S14–S17). Tetracycline chemotherapeutics can undergo conformational changes at different pH or when bound to Mg^2+^ ions, possibly influencing the overall CD.^57−60^ Therefore, the observed CD changes can possibly result from either dsRNA and tetracycline structural changes or a combination of effects from the dsRNA structure and the tetracycline–metal complexation. In the future, advanced techniques, such as CD thermal analysis, could be employed to address these questions.

**Figure 4:**
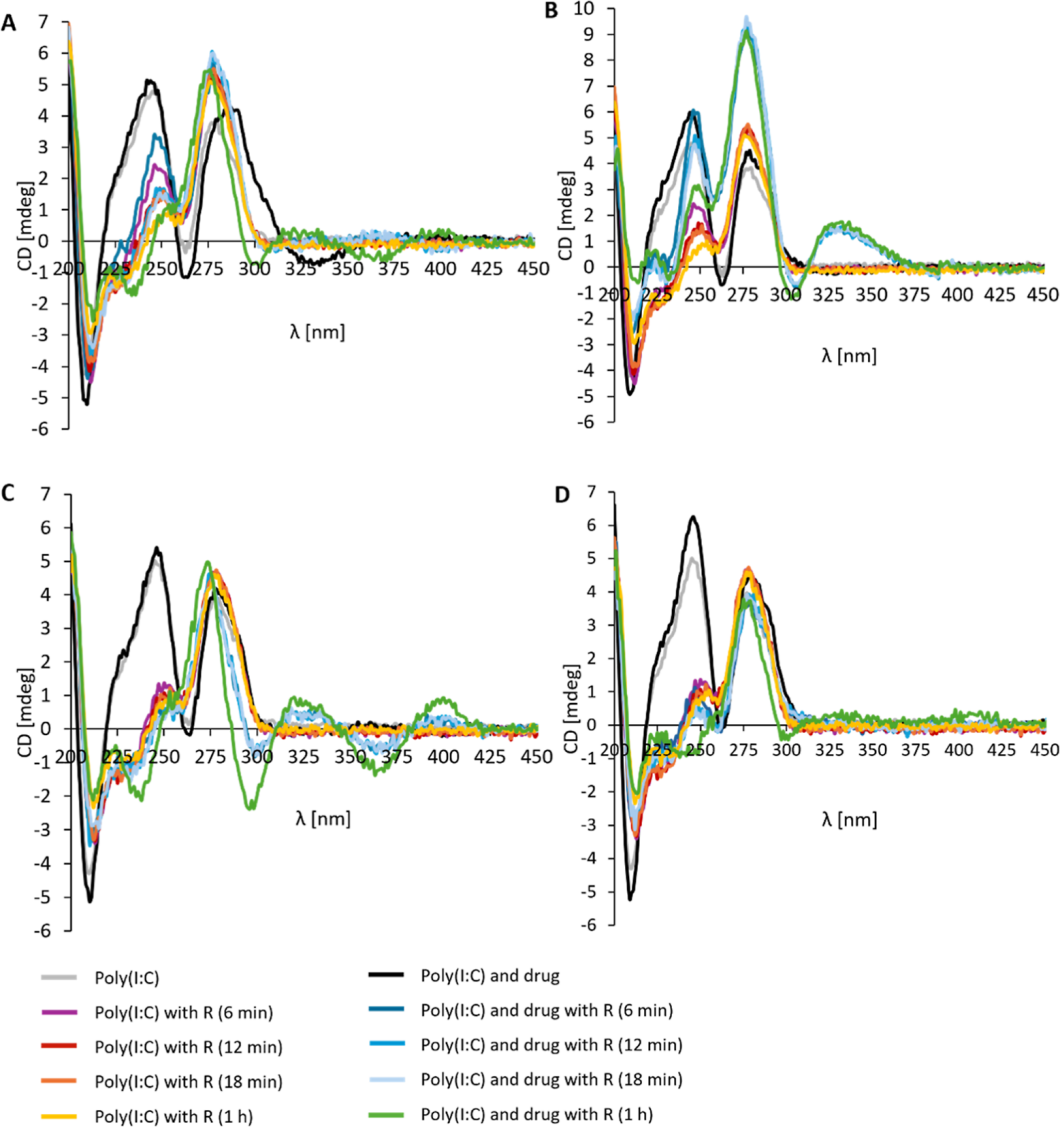
(A) Effect of RNase on the CD spectra of Poly(I:C) HMW alone and with doxycycline hyclate in the absence of 10 mM MgSO_4_ (B) and in the presence of 10 mM MgSO_4_. (C) Effect of RNase on the CD spectra of Poly(I:C) HMW alone and with minocycline hydrochloride in the absence of 10 mM MgSO_4_ (D) and in the presence of 10 mM MgSO_4_. The concentration of Poly(I:C)s and chemotherapeutics was 1 × 10^–4^ M (1:1).

**Figure 5:**
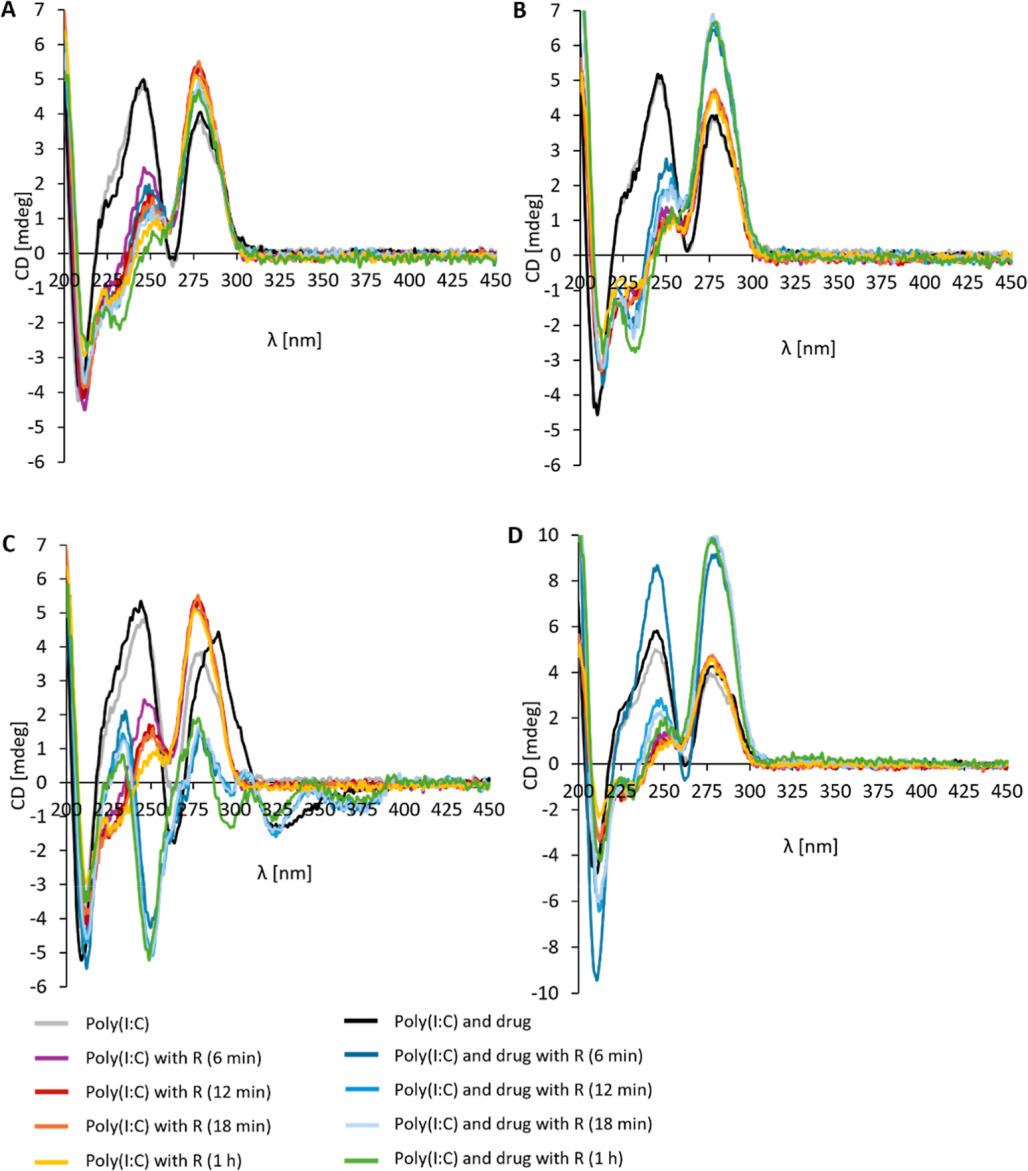
(A) Effect of RNase on the CD spectra of Poly(I:C) HMW alone and with doxorubicin hydrochloride in the absence of 10 mM MgSO_4_ (B) and in the presence of 10 mM MgSO_4_. (C) Effect of RNase on the CD spectra of Poly(I:C) HMW alone and with tetracycline hydrochloride in the absence of 10 mM MgSO_4_ (D) and in the presence of 10 mM MgSO_4_. The concentration of Poly(I:C)s and chemotherapeutics was 1 × 10^–4^ M (1:1).

To study the protective effect of chemotherapeutics against the nuclease cleavage of Poly(I:C)s, RNase III was added to the samples. The incubation of all Poly(I:C)s with RNase III decreased the intensity of CD spectra at around 245 nm, indicating the degradation of the dsRNA structure (Figures 4 and 5, S10–S13). Although the affinity of chemotherapeutics for Poly(I:C) molecules was suggested, the nuclease effectively hydrolyzed all Poly(I:C)s in the presence of tetracycline (Figures 5C,D and S10–13D) and doxorubicin (Figures 5A,B and S10–13C), independently of MgSO_4_. The ellipticity of Poly(I:C) Na^+^ (Figure S10B) and Poly(I:C) Na^+^ γ (Figure S11B) decreased slowly after the addition of RNase III in the presence of minocycline and Poly(I:C) HMW in the presence of doxycycline (Figure 4A), demonstrating the protection of Poly(I:C)s in the absence of MgSO_4_. Similar results were also observed by Chukwudi and Good.^21^ However, the CD intensity decreased eventually, showing partial protection of Poly(I:C) molecules against nuclease degradation. In the case of tetracycline, a negative band appeared around 250 nm in the absence of MgSO_4_ (Figures 5C and S10–11D). The presence of MgSO_4_ did not further protect Poly(I:C) molecules from the RNase activity. It can be speculated that the addition of MgSO_4_ to the RNase reaction buffer might be a double-edged sword as Mg^2+^ ions are also essential for RNase activity.^61^ To further analyze the potential protection of Poly(I:C) molecules against nuclease cleavage, CD intensity values were normalized to the initial measurements without RNase at the maximum wavelength band intensity for each Poly(I:C)–chemotherapy combination and analyzed using nonlinear regression (Figure S18). The rate constant (*K*) and half-life (*t*_1/2_) were determined and indicate prolonged half-life for Poly(I:C) Na^+^ γ and doxycycline, Poly(I:C) HMW and doxycycline, and all Poly(I:C)s and minocycline compared to the Poly(I:C) molecule alone after the addition of RNase III enzyme (Table S1). In the presence of 10 mM MgSO_4_, prolonged half-life was suggested for Poly(I:C) Na^+^ and minocycline, Poly(I:C) HMW and minocycline, Poly(I:C) HMW and doxorubicin, and Poly(I:C) HMW and tetracycline.

### Microscale Thermophoresis

**Figure 6:**
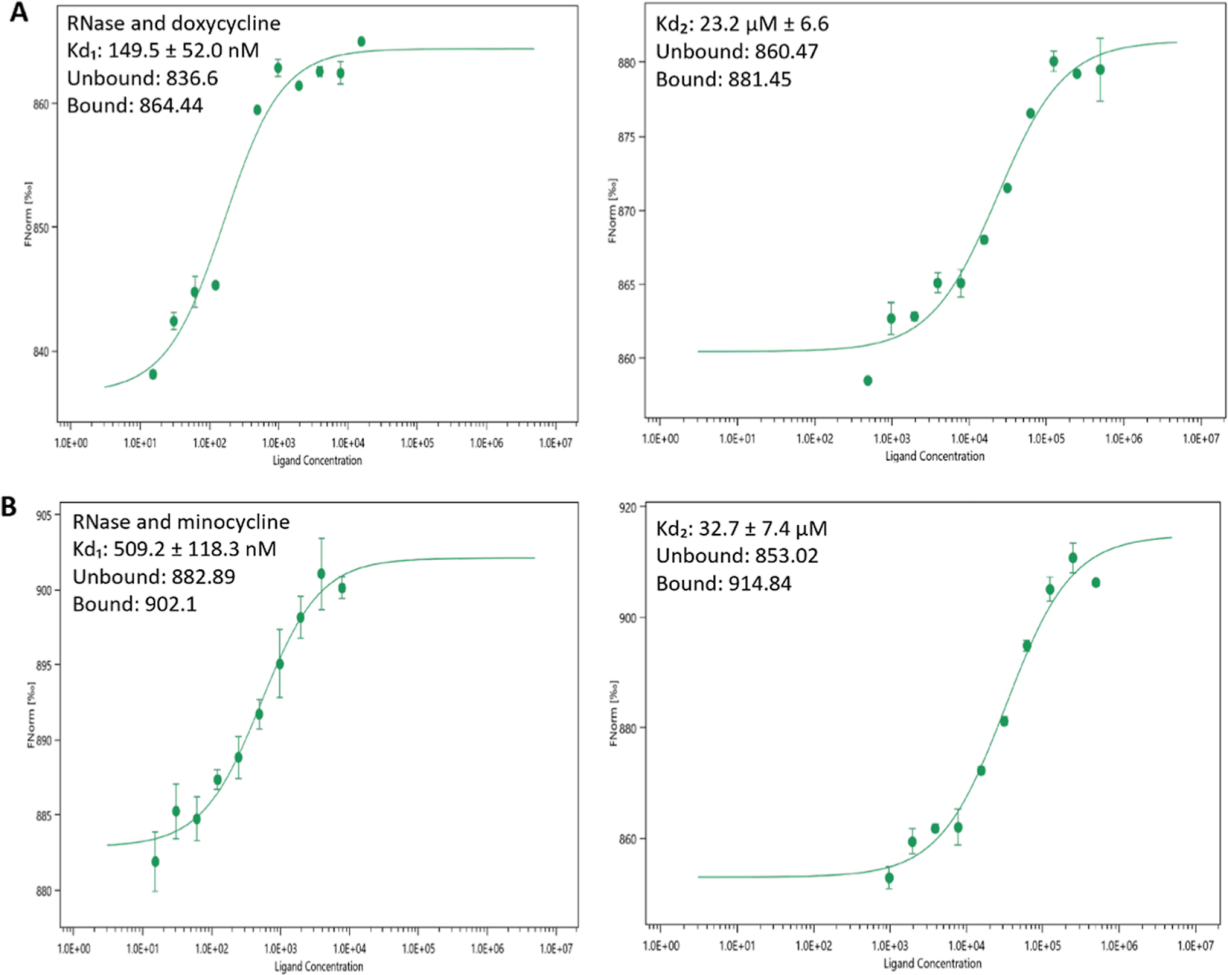
Binding of (A) doxycycline and (B) minocycline to a human RNase, and *K*_d_ values determined from the MST data.

To experimentally support in silico docking results, we performed microscale thermophoresis (MST). All the selected chemotherapeutics were titrated against a fluorescently labeled Poly(I:C) HMW [Poly(I:C) HMW fluorescein] and his-tag-labeled human RNase (Figures 6 and S20). After the measurements, MST data were evaluated. Both temperature jump, defined as a change in fluorescence intensity before the heat-induced migration of measured molecules, and thermophoresis, the heat induced-migration, allow for the determination of dissociation constant *K*_d_. Therefore, we processed the temperature jump and thermophoresis. *K*_d_ values were calculated from a fluorescence–ligand concentration fitted curve. For measurements with RNase and doxycycline/minocycline, two dissociation constants were determined, revealing two possible binding sites (Figure 6). Dissociation constants for RNase and chemotherapy were as follows: *K*_d_ = 4.8 ± 2 μM for doxorubicin, *K*_d1_ = 149.5 ± 52.0 nM, and *K*_d2_ = 23.2 ± 6.6 μM for doxycycline, *K*_d1_ = 509.2 ± 118.3 nM and *K*_d2_ = 32.7 ± 7.4 μM for minocycline, and *K*_d_ = 24.5 μM for tetracycline. Dissociation constants for Poly(I:C) HMW with doxorubicin, doxycycline, minocycline, and tetracycline were as follows: *K*_d_ 251 ± 22 μM, *K*_d_ 366.2 ± 74.9 μM, *K*_d_ 393.2 ± 860.1 μM, and *K*_d_ 448.1 ± 86.4 μM.

### Dual NF-κB and IRF Assay

**Figure 7:**
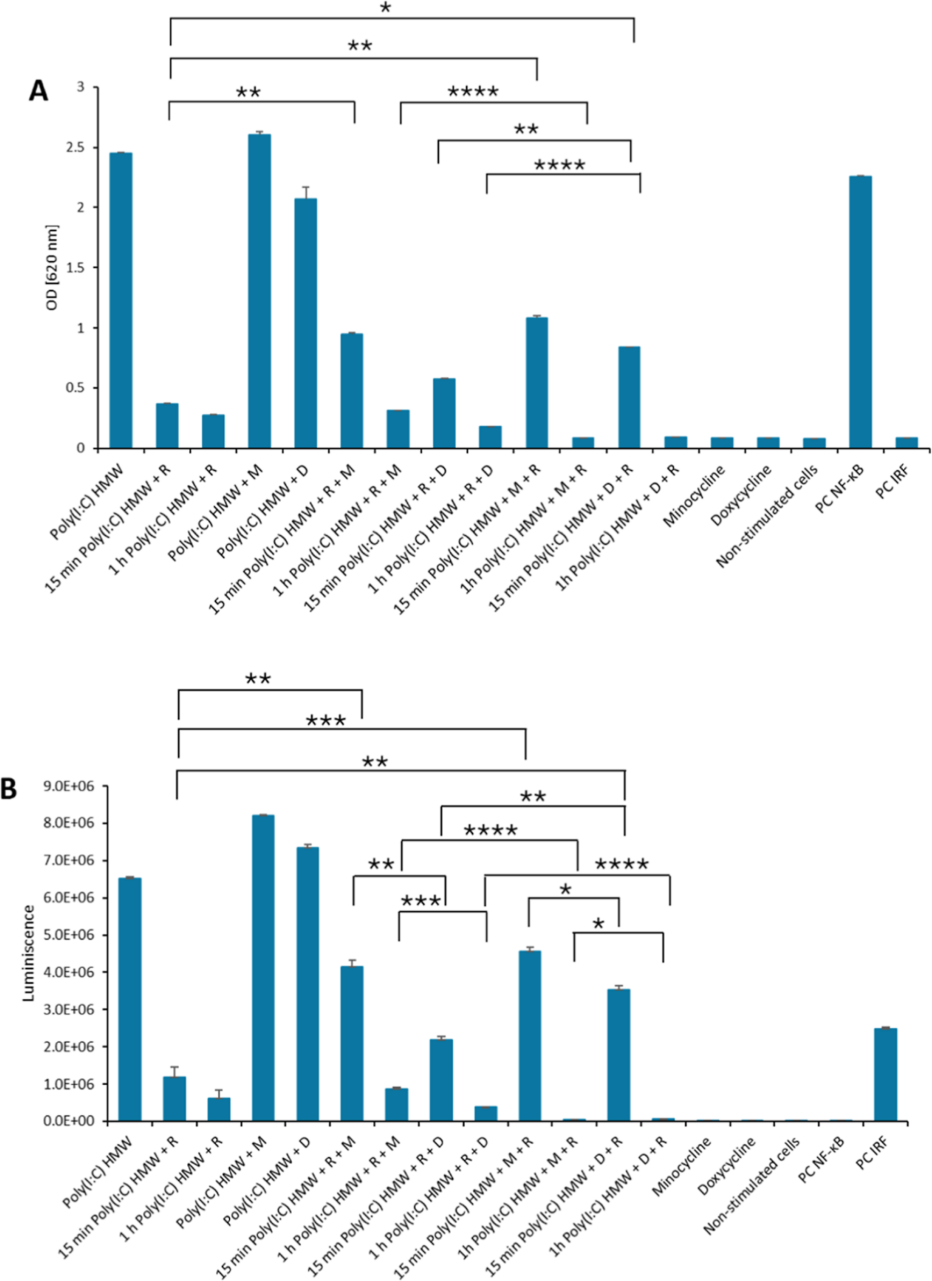
(A) NF-κB response of HEK dual hTLR3 cells to treatment with Poly(I:C) HMW + minocycline/doxycycline + RNase and Poly(I:C) HMW + RNase + minocycline/doxycycline. (B) Activation of IRF pathway upon Poly(I:C) HMW + minocycline/doxycycline + RNase and Poly(I:C) HMW + RNase + minocycline/doxycycline. Data are presented as mean ± SEM. *P*-values ≤ were considered statistically significant (*≤ 0.5; **≤ 0.01; ***≤ 0.001; ****≤ 0.0001).

Minocycline and doxycycline hyclate were consequently selected for the biological evaluation of the potential protection of Poly(I:C) molecules against RNase degradation *in vitro*. HEK dual hTLR3 cells were selected for such a purpose as described elsewhere.^62^ They are HEK293-derived cell lines that allow quantification of the activation of NF-κB and IRF pathways mediated via the interaction of Poly(I:C) with Toll-like receptor 3 (TLR3). Prior to the dual NF-κB and IRF assay, a cytotoxic assay of chemotherapeutics was performed in order to select concentrations with no cytotoxicity. After 48 h, the viability of the cells at 1–2 μM concentration was around 90% (Figure S21). Therefore, concentrations of 1 μM (doxycycline) and 2 μM (minocycline) and incubation time of 24 h were selected for the dual assay. Very low activation of NF-κB and IRF was observed for all Poly(I:C) Na^+^ and Poly(I:C) Na^+^ γ samples, either with doxycycline or minocycline, indicating their complete cleavage by the nuclease (Figure S22). In the case of Poly(I:C) HMW, doxycycline and minocycline prolonged the protection of the Poly(I:C) HMW molecule. Higher NF-κB and IRF responses were detected for Poly(I:C)-minocycline/doxycycline samples incubated for 15 min with RNase compared to the cleaved Poly(I:C) sample alone. Interestingly, similar NF-κB response levels were found for previously treated Poly(I:C) HMW molecules with RNase, followed by the addition of minocycline/doxycycline. These results can be explained by the preserved biological activity of cleaved long double strands of high-molecular Poly(I:C) and the limited ability of minocycline and doxycycline to activate the pathway (Figure 7).

## Conclusions

In this study, we combined commercial immunostimulatory Poly(I:C) molecules and oncolytic anthracycline and tetracycline chemotherapeutics to investigate their possible interactions and protective abilities against the nuclease degradation of Poly(I:C)s. Fluorescence spectroscopy revealed interactions between all dsRNA analogues with selected chemotherapeutics. The lowest binding affinity was found for Poly(I:C) Na^+^ γ, probably due to the sample heterogeneity and lower stability induced by the irradiation of the strands. Supplementation with MgSO_4_ led to an increase in the binding affinity and melting temperatures of Poly(I:C)s. In silico docking data and microscale thermophoresis suggested a binding interaction of chemotherapeutics to the RNase–Poly(I:C) complex as a mechanism of prolonged resistance to nuclease degradation. The ability of minocycline and doxycycline to partially protect the cleavage of Poly(I:C) was revealed by a nonlinear regression of CD spectra and further observed for minocycline and doxycycline *in vitro*. Our results indicate that combining commercial Poly(I:C) molecules with minocycline and doxycycline may prolong the duration of Poly(I:C)’s biological activity. We also suggest that this chemoimmunotherapy approach may allow lower therapeutic doses, decrease potential side effects when administered intratumorally, or exert synergy in animal cancer models. However, to draw such conclusions, further research is necessary.

## Supporting Information Available

Experimental procedures, additional experimental methods, and results (DOCX)

## Abbreviations

SR/CR: spontaneous regression/complete resistant
DNA: deoxyribonucleic acid
mRNA: messenger RNA.
