# Supplementary material for: Interaction of Selected Anthracycline and Tetracycline Chemotherapeutics with Poly(I:C) Molecules
Source: ACS Omega. 2025 Apr 15;10(16):15935–46. doi: 10.1021/acsomega.4c05483 (PMC12044458; doi:10.1021/acsomega.4c05483)
Supplement: Supplementary file 1 — ao4c05483_si_001.docx [file ao4c05483_si_001.docx]

Supporting Information

**Interaction of Selected Anthracycline and Tetracycline Chemotherapeutics with**

**Poly(I:C) Molecules**

Markéta Skaličková^1,2^, Nikita Abramenko^1,2^, Tatsiana Charnavets^3^, Frédéric Vellieux^1,2^, Jindřiška Leischner Fialová^1^, Kateřina Kučnirová^1,2^, Zdeněk Kejík^1,2^, Michal Masařík^1,2,4,5^, Pavel Martásek^2^, Karel Pacak^6^, Tomáš Pacák^7^, and Milan Jakubek^1,2*^

*^1^BIOCEV, First Faculty of Medicine, Charles University, 252 50 Vestec, Czech Republic*

*^2^Department of Paediatrics and Inherited Metabolic Disorders, First Faculty of Medicine, Charles University and General University Hospital, 120 00 Prague, Czech Republic*

*^3^Institute of Biotechnology of the Czech Academy of Sciences, BIOCEV, Vestec, Czech Republic*

*^4^Department of Physiology, Faculty of Medicine, Masaryk University, Kamenice 5, 625 00 Brno, Czech Republic*

*^5^Department of Pathological Physiology, Faculty of Medicine, Masaryk University, Kamenice 5, CZ-625 00 Brno, Czech Republic*

*^6^Section on Medical Neuroendocrinology, Eunice Kennedy Shriver National Institute of Child Health and Human Development, National Institutes of Health, Building 10, Room 1‑3140, 10 Center Drive, Bethesda, MD 20892, USA*

*^7^TumorSHOT, Italská 2581/67, Vinohrady, Praha 2, 120 00 Prague, Czech Republic*

* Correspondence: [Milan.Jakubek@lf1.cuni.cz](mailto:Milan.Jakubek@lf1.cuni.cz)

Table of Contents

[**1** **RESULTS** S3](#_Toc190170257)

[**1.1** **Stability of Selected Poly(I:C) Molecules** S3](#_Toc190170258)

[**1.2** **Conditional Binding Constant and Stoichiometry between Chemotherapeutics and Poly(I:C) Molecules** S5](#_Toc190170259)

[**1.3** **In Silico Docking of Chemotherapeutics (and of Poly(I:C)) to a Human Nuclease** S10](#_Toc190170260)

[**1.4** **Thermal Stability of RNase III** S11](#_Toc190170261)

[**1.5** **Circular Dichroism of Poly(I:C) Molecules with Chemotherapeutics** S12](#_Toc190170262)

[**1.6** **Microscale Thermophoresis of Chemotherapeutics to a Human Nuclease and Poly(I:C) Molecule** S22](#_Toc190170263)

[**1.7** **Cytotoxicity Assay of Minocycline and Doxycycline** S24](#_Toc190170264)

[**1.8** **Dual NF-κB and IRF Assay** S24](#_Toc190170265)

[**2** **MATERIALS AND METHODS** S25](#_Toc190170266)

[**2.1** **Materials** S25](#_Toc190170267)

[**2.2** **Stability of Selected Poly(I:C) Molecules** S26](#_Toc190170268)

[**2.3** **Conditional Binding Constant and Stoichiometry between Chemotherapeutics and Poly(I:C) Molecules** S26](#_Toc190170269)

[**2.4** **UV-Vis Melting of Poly(I:C)-chemotherapeutic Complexes** S27](#_Toc190170270)

[**2.5** **In Silico Docking of Chemotherapeutics (and of Poly(I:C)) to a Human Nuclease** S27](#_Toc190170271)

[**2.6** **Thermal Stability of RNase III** S28](#_Toc190170272)

[**2.7** **Circular Dichroism of Poly(I:C) Molecules with Chemotherapeutics** S28](#_Toc190170273)

[**2.8** **Microscale Thermophoresis of Chemotherapeutics to a Human Nuclease and Poly(I:C) Molecule** S28](#_Toc190170274)

[**2.9** **Cytotoxicity Assay of Minocycline and Doxycycline** S29](#_Toc190170275)

[**2.10** **Dual NF-κB and IRF Assay** S30](#_Toc190170276)

[**3** **REFERENCES** S31](#_Toc190170277)

# **RESULTS**

## **Stability of Selected Poly(I:C) Molecules**


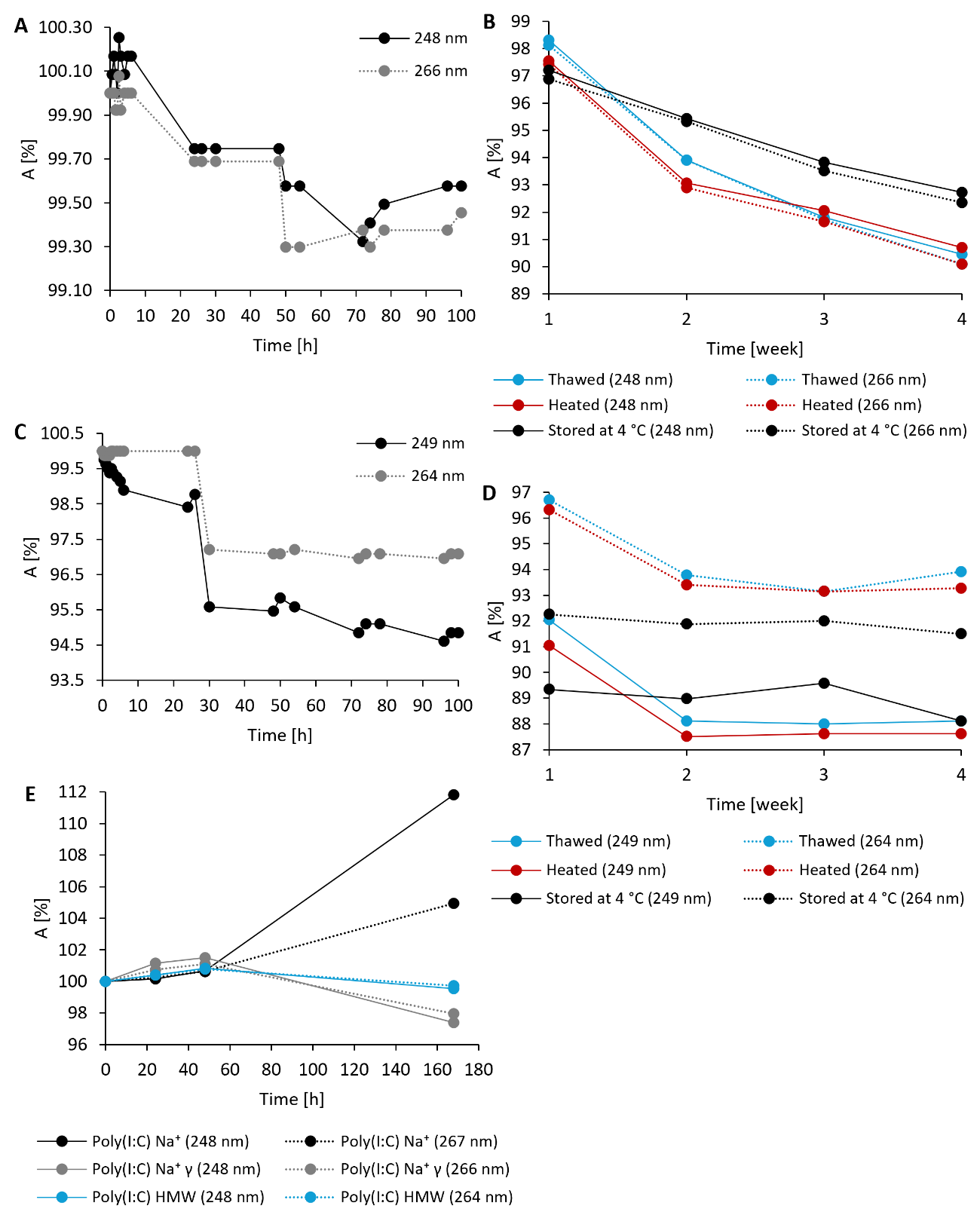


Figure S1: **(A)** Absorbance changes (%) of Poly(I:C) Na⁺ at absorbance maxima 248 and 266 nm for up to 100 hours at 4 °C in 0.9% NaCl (pH 5.8) (compared to absorbance at time 0h (A_0h_)). **(B)** Absorbance changes (%) of Poly(I:C) Na⁺ at the absorbance maxima 248 and 266 nm after storage at 4 °C, repeated freezing (-20 °C), and thawing (50 °C) for one month in 0.9% NaCl (pH 5.8) (compared to A_0h_ stored at 4 °C. **(C)** Absorbance changes (%) of Poly(I:C) Na⁺ γ at absorbance maxima 249 and 264 nm for up to 100 hours at 4 °C in 0.9% NaCl (pH 5.8) (compared to absorbance at time 0h (A_0h_)). **(D)** Absorbance changes (%) of Poly(I:C) Na⁺ γ at the absorbance maxima 249 and 264 nm after storage at 4 °C, repeated freezing (-20 °C), and thawing (50 °C) for one month in 0.9% NaCl (pH 5.8) (compared to A_0h_ stored at 4 °C). **(E)** Absorbance changes (%) of Poly(I:C) Na^+^ (at 248 and 267 nm) Poly(I:C) HMW (at 248 and 264 nm), and Poly(I:C) Na^+^ γ (at 248 and 266 nm)(100 μg/mL) at the absorbance maxima in PBS for one week (compared to A_0h_).

## **Conditional Binding Constant and Stoichiometry between Chemotherapeutics and Poly(I:C) Molecules**


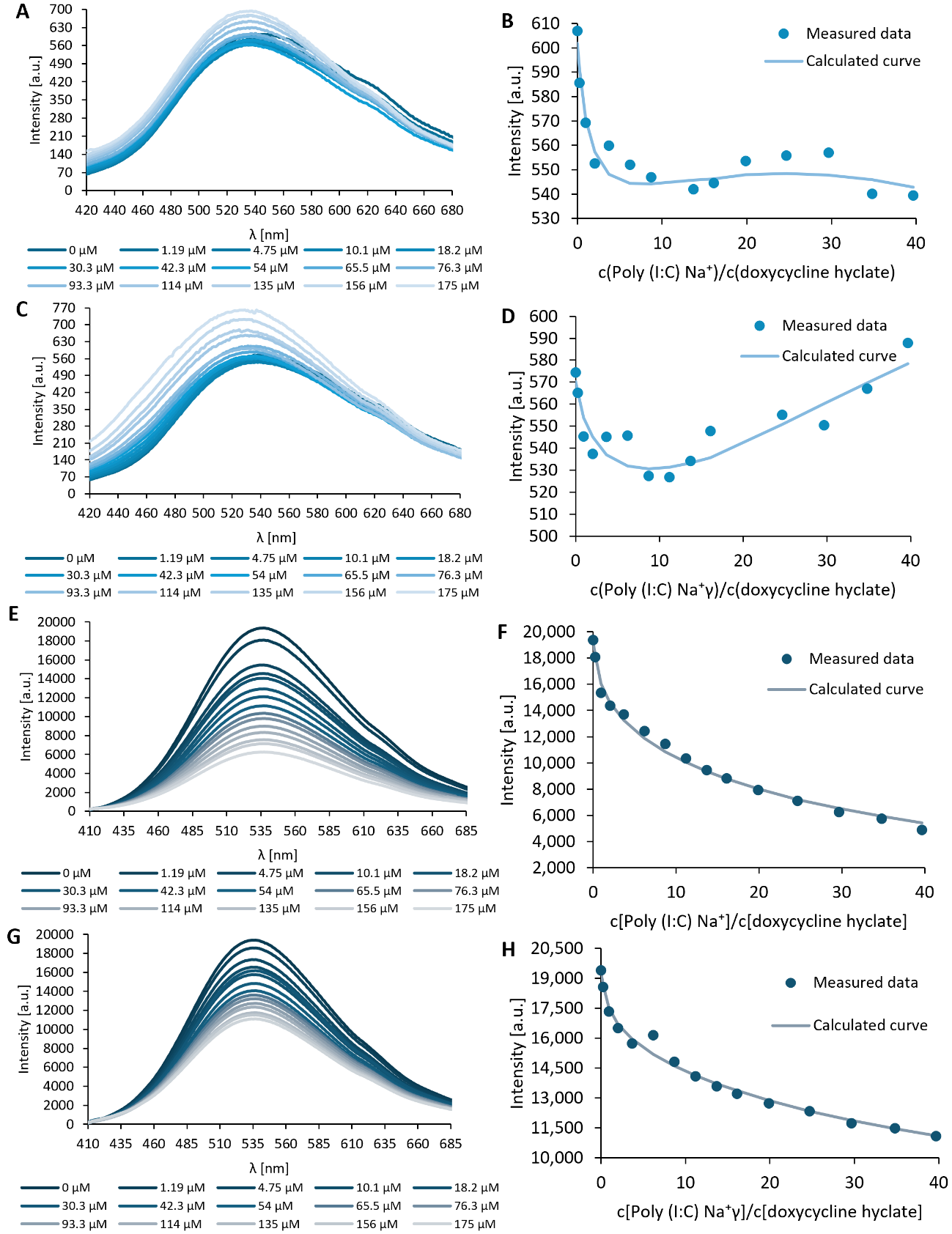


Figure S2: **(A)** Fluorescence emission spectrum and **(B)** fluorescence curve of doxycycline hyclate at 540 nm in the absence and presence of increasing amounts of Poly(I:C) Na^+^ in PBS. **(C)** Fluorescence emission spectrum and **(D)** fluorescence curve of doxycycline hyclate at 540 nm in the absence and presence of increasing amounts of Poly(I:C) Na^+^ γ in PBS. **(E)** Fluorescence emission spectrum and **(F)** fluorescence curve of doxycycline hyclate at 537 nm in the absence and presence of increasing amounts of Poly(I:C) Na^+^in the presence of 10 mM MgSO_4_ in PBS. **(G)** Fluorescence emission spectrum and **(H)** fluorescence curve of doxycycline hyclate at 537 nm in the absence and presence of increasing amounts of Poly(I:C) Na^+^ γ in the presence of 10 mM MgSO_4_ in PBS. The concentration of each chemotherapeutic agent was held constant at 5×10^-6^ M. The molar concentration of Poly(I:C) varied in the range of 1.2×10^-6^ to 1.75×10^-4^ M.


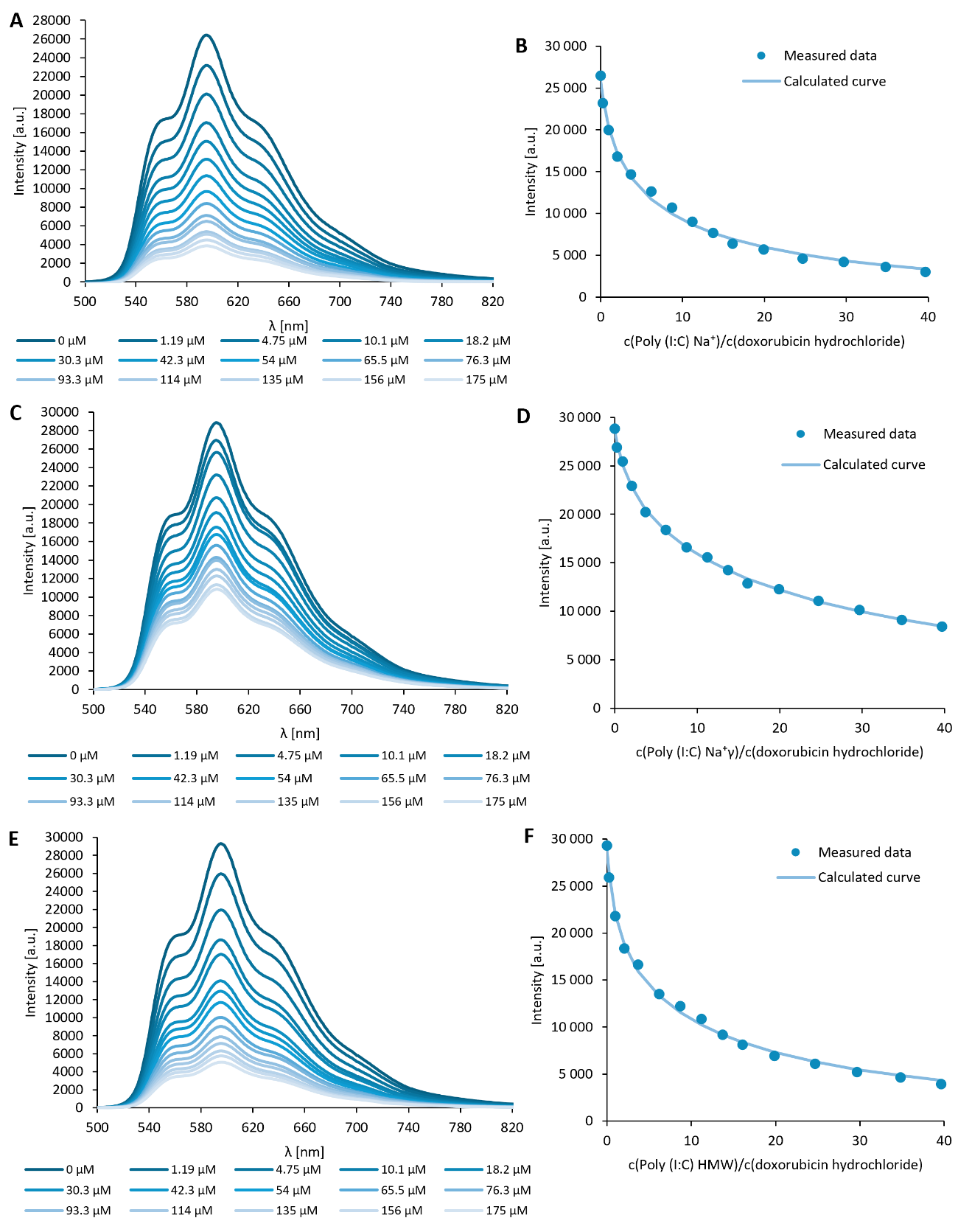


Figure S3: **(A)** Fluorescence emission spectrum and **(B)** fluorescence curve of doxorubicin hydrochloride at 595 nm in the absence and presence of increasing amounts of Poly(I:C) Na^+^in PBS. **(C)** Fluorescence emission spectrum and **(D)** fluorescence curve of doxorubicin hydrochloride at 595 nm in the absence and presence of increasing amounts of Poly(I:C) Na^+^ γ in PBS. **(E)** Fluorescence emission spectrum and **(F)** fluorescence curve of doxorubicin hydrochloride at 595 nm in the absence and presence of increasing amounts of Poly(I:C) HMW in PBS. The concentration of each chemotherapeutic agent was held constant at 5×10^-6^ M. The molar concentration of Poly(I:C) varied in the range of 1.2×10^-6^ to 1.75×10^-4^ M.


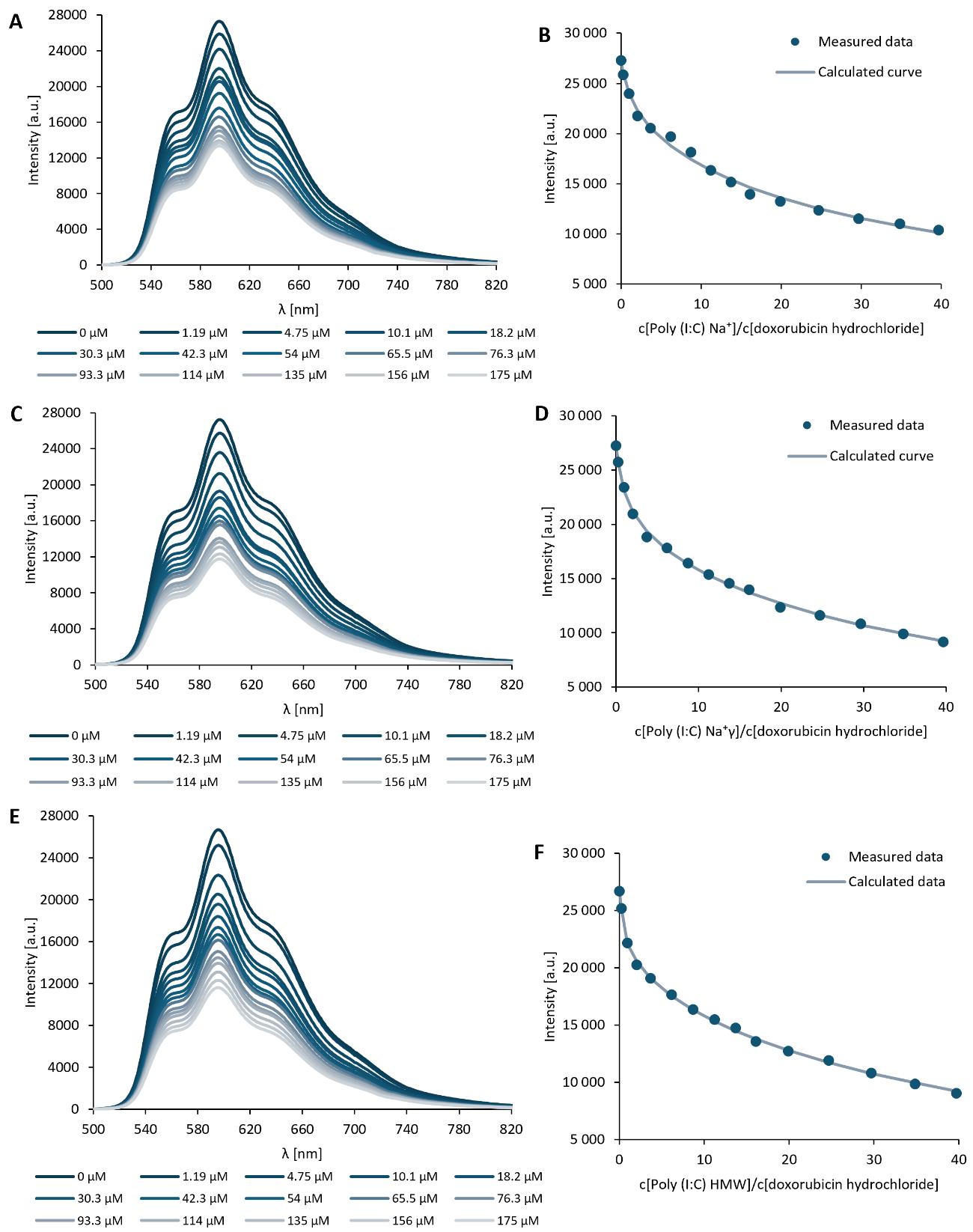


Figure S4: **(A)** Fluorescence emission spectrum and **(B)** fluorescence curve of doxorubicin hydrochloride at 595 nm in the absence and presence of increasing amounts of Poly(I:C) Na^+^in the presence of 10 mM MgSO_4_ in PBS. **(C)** Fluorescence emission spectrum and **(D)** fluorescence curve of doxorubicin hydrochloride at 595 nm in the absence and presence of increasing amounts of Poly(I:C) Na^+^ γ in the presence of 10 mM MgSO_4_ in PBS. **(E)** Fluorescence emission spectrum and **(F)** fluorescence curve of doxorubicin hydrochloride at 595 nm in the absence and presence of increasing amounts of Poly(I:C) HMW in the presence of 10 mM MgSO_4_ in PBS. The concentration of each chemotherapeutic agent was held constant at 5×10^-6^ M. The molar concentration of Poly(I:C) varied in the range of 1.2×10^-6^ to 1.75×10^-4^ M.


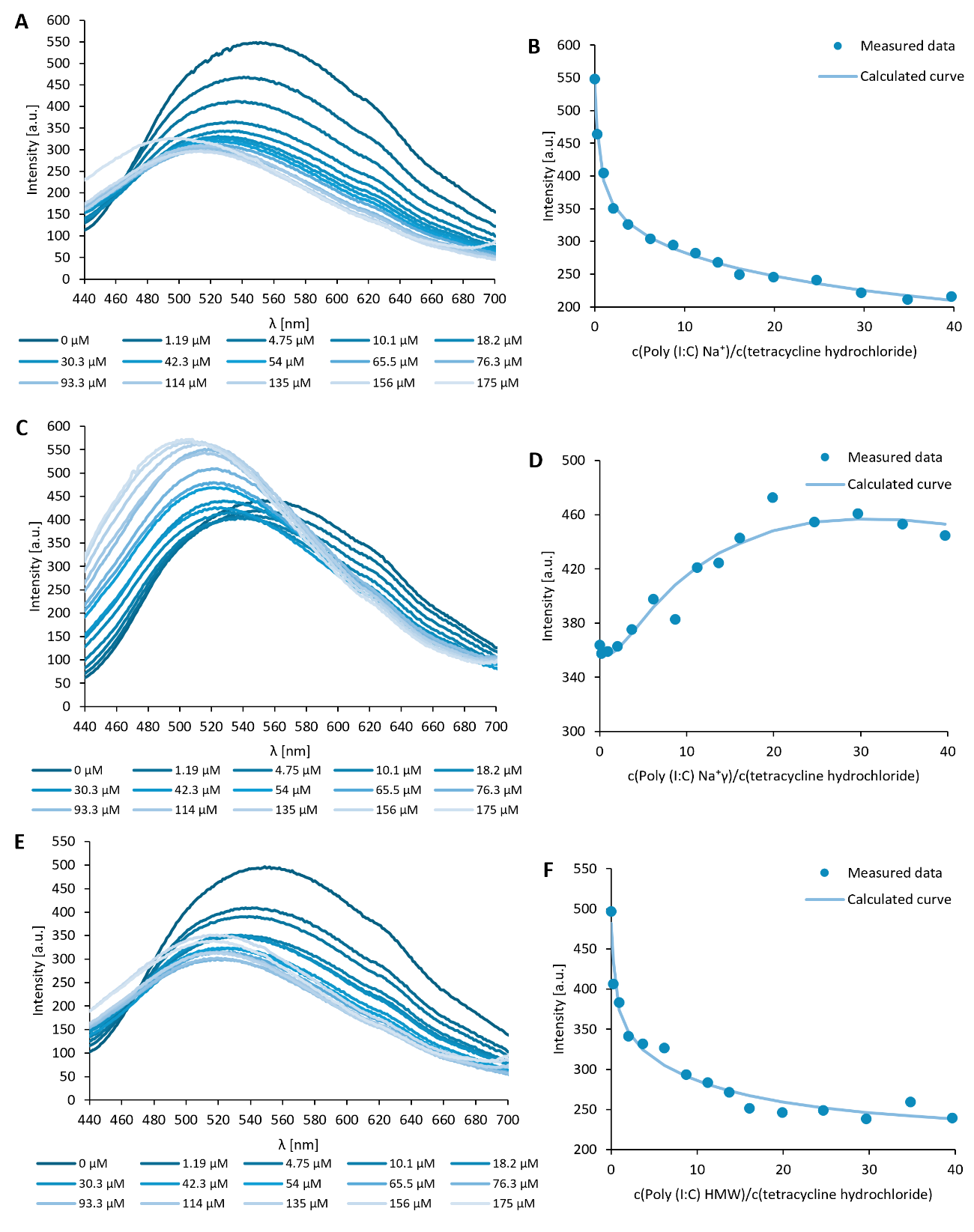


Figure S5: **(A)** Fluorescence emission spectrum and **(B)** fluorescence curve of tetracycline hydrochloride at 549 nm in the absence and presence of increasing amounts of Poly(I:C) Na^+^ in PBS. **(C)** Fluorescence emission spectrum and **(D)** fluorescence curve of tetracycline hydrochloride at 549 nm in the absence and presence of increasing amounts of Poly(I:C) Na^+^ γ in PBS. **(E)** Fluorescence emission spectrum and **(F)** fluorescence curve of tetracycline hydrochloride fluorescence at 549 nm in the absence and presence of increasing amounts of Poly(I:C) HMW in PBS. The concentration of each chemotherapeutic agent was held constant at 5×10^-6^ M. The molar concentration of Poly(I:C) varied in the range of 1.2×10^-6^ to 1.75×10^-4^ M.


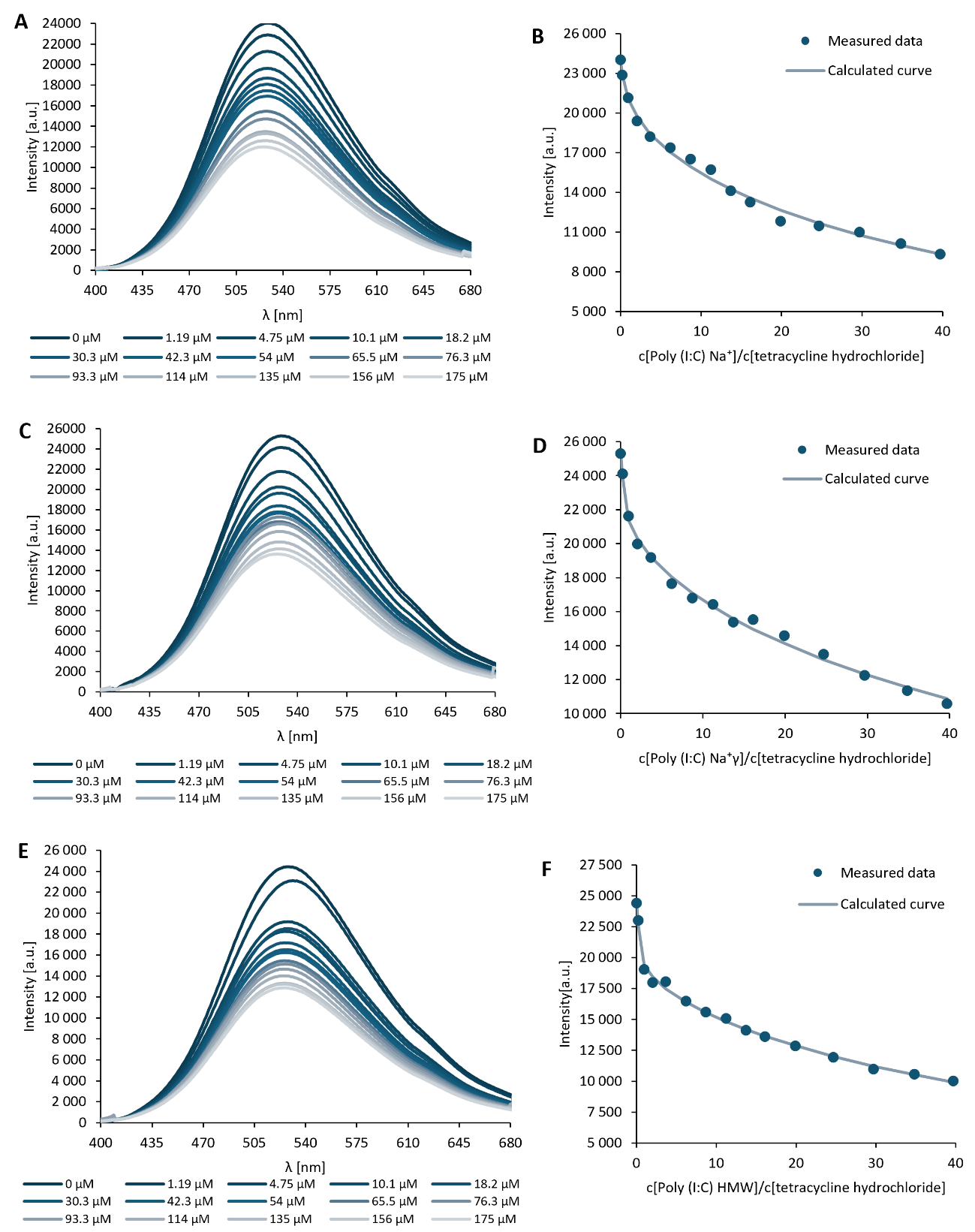


Figure S6: **(A)** Fluorescence emission spectrum and **(B)** fluorescence curve of tetracycline hydrochloride at 528 nm in the absence and presence of increasing amounts of Poly(I:C) Na^+^in the presence of 10 mM MgSO_4_ in PBS. **(C)** Fluorescence emission spectrum and **(D)** fluorescence curve of tetracycline hydrochloride at 528 nm in the absence and presence of increasing amounts of Poly(I:C) Na^+^ γ in the presence of 10 mM MgSO_4_ in PBS. **(E)** Fluorescence emission spectrum and **(F)** fluorescence curve of tetracycline hydrochloride at 528 nm in the absence and presence of increasing amounts of Poly(I:C) HMW in the presence of 10 mM MgSO_4_ in PBS. The concentration of each chemotherapeutic agent was held constant at 5×10^-6^  M. The molar concentration of Poly(I:C) varied in the range of 1.2×10^-6^ to 1.75×10^-4^ M.

## **In Silico Docking of Chemotherapeutics (and of Poly(I:C)) to a Human Nuclease**


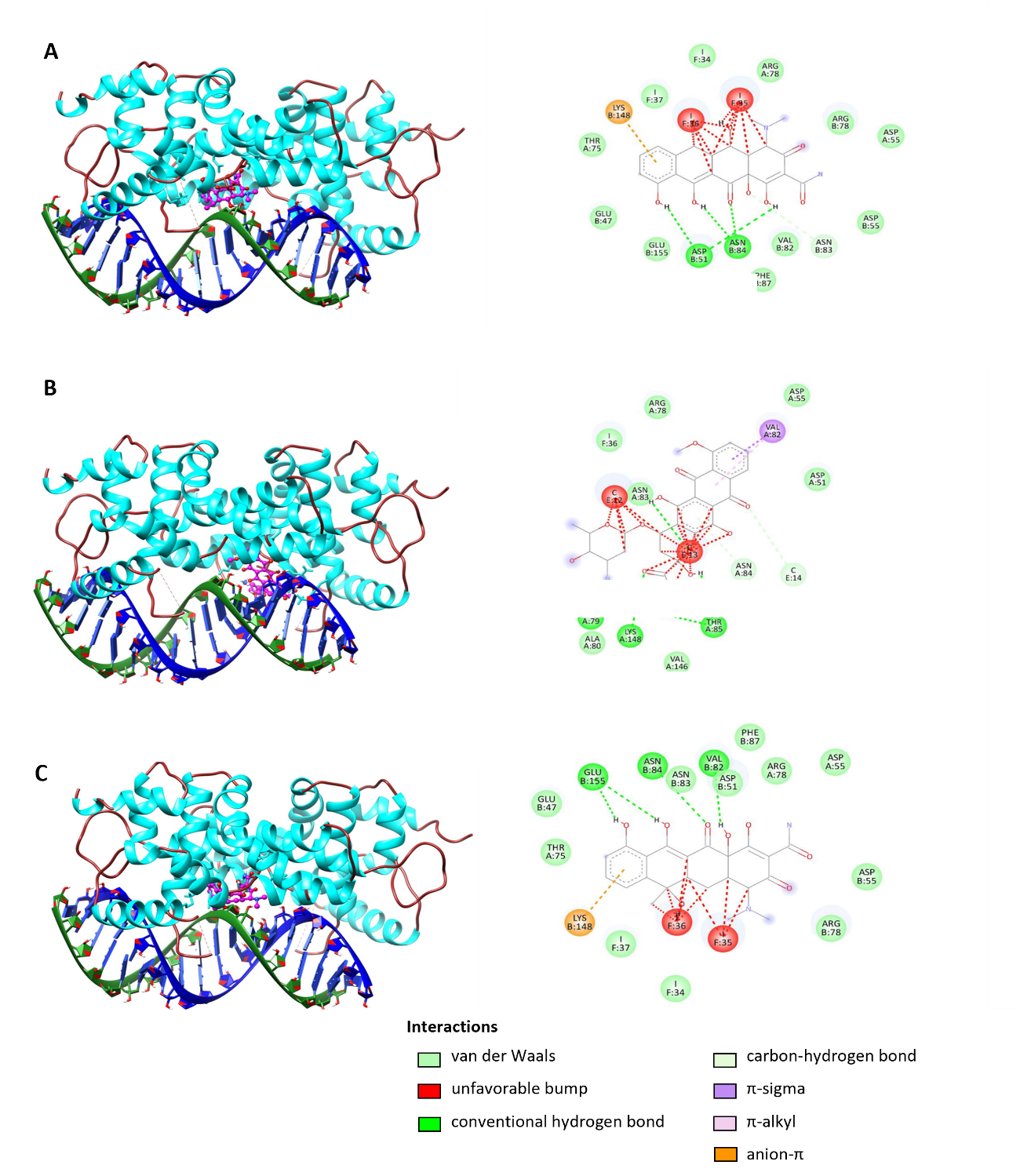


Figure S7: General view of the docked position of **(A)** doxycycline, **(B)** doxorubicin, and **(C)** tetracycline with the RIIID-Poly(I:C) complex (left) and 2D diagram of the interactions (right).

## **Thermal Stability of RNase III**

The thermal stability of Ambion RNase III in PBS was determined for circular dichroism experiments. The measured temperature was 56.9 °C (Figure S8).


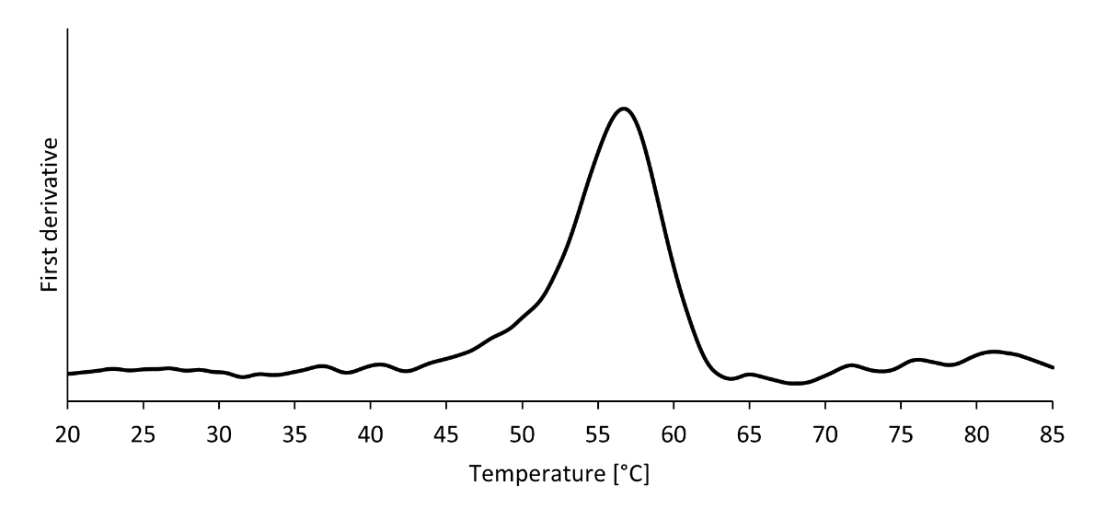


Figure S8: Thermal stability of Ambion RNase III in PBS.

## **Circular Dichroism of Poly(I:C) Molecules with Chemotherapeutics**


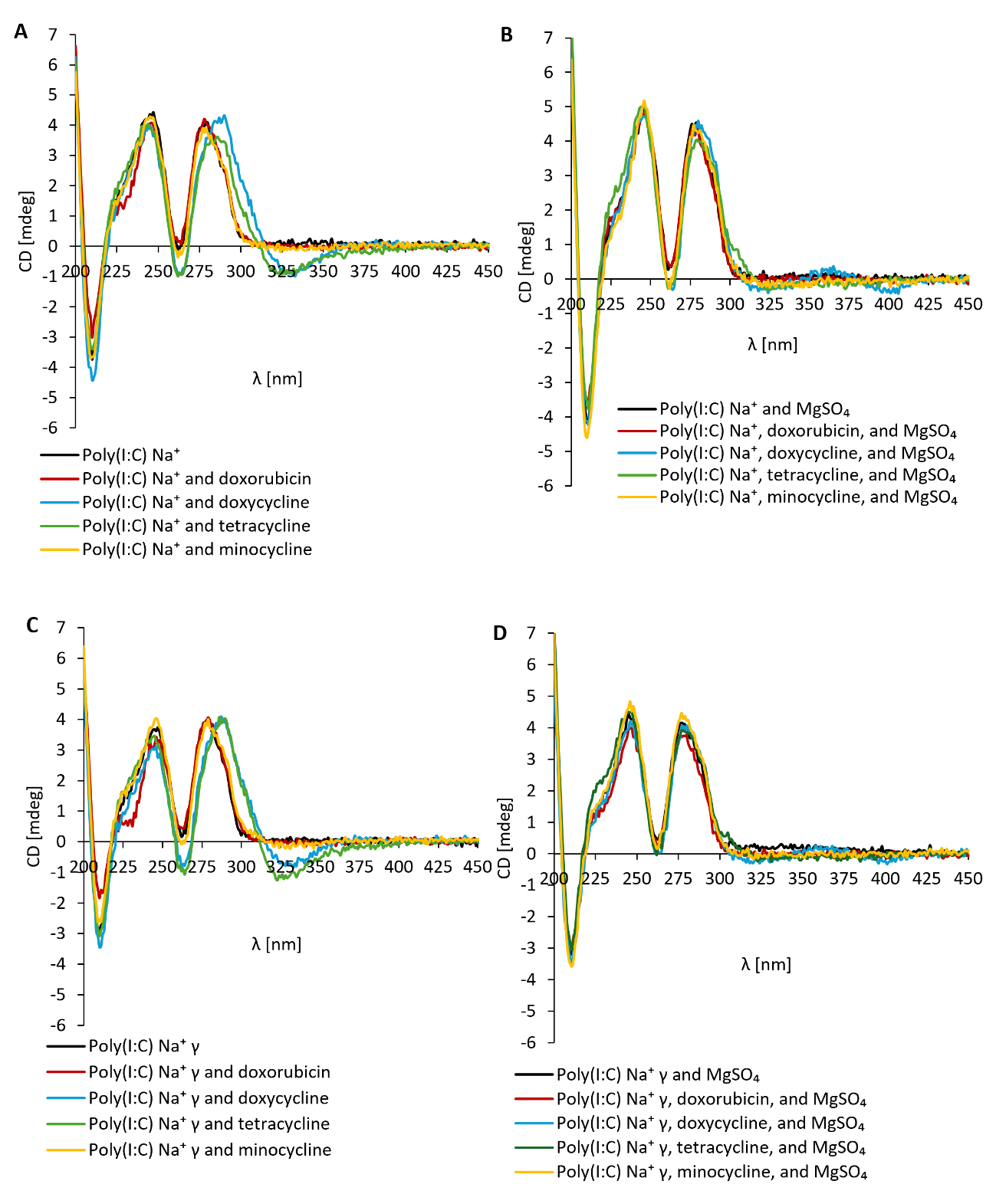


Figure S9: **(A)** Circular dichroism spectra of chemotherapeutics, Poly(I:C) Na⁺, and their combinations in the absence of 10 mM MgSO_4_ and **(B)** presence of 10 mM MgSO_4_ in PBS. **(C)** Circular dichroism spectra of chemotherapeutics, Poly(I:C) Na⁺ γ, and their combinations in the absence of 10 mM MgSO_4_ and **(D)** presence of 10 mM MgSO_4_ in PBS.


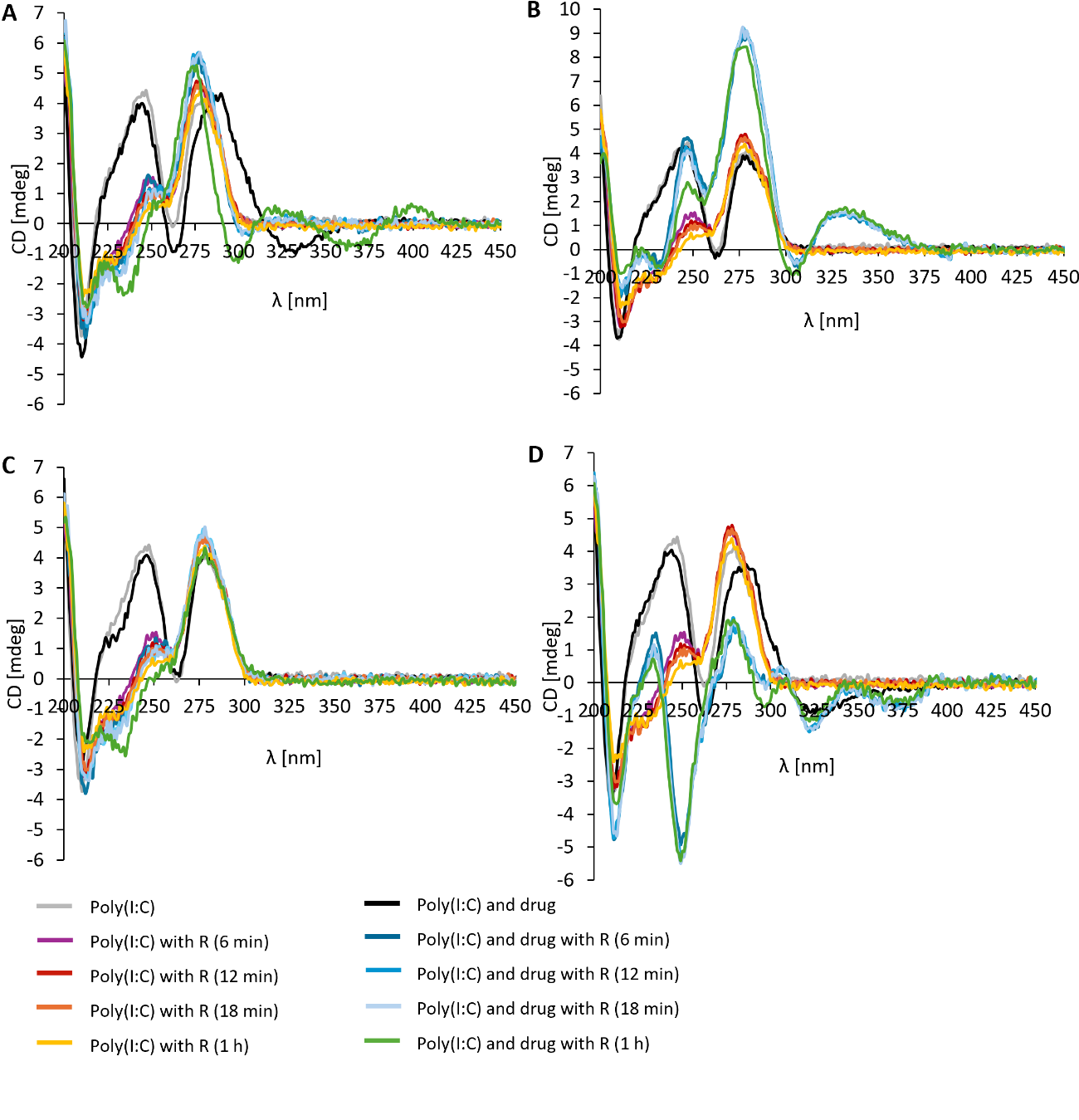


Figure S10: Effect of RNase on CD spectra of Poly(I:C) Na⁺ alone and with **(A)** doxycycline, **(B)** minocycline, **(C)** doxorubicin, **(D)** tetracycline in PBS.


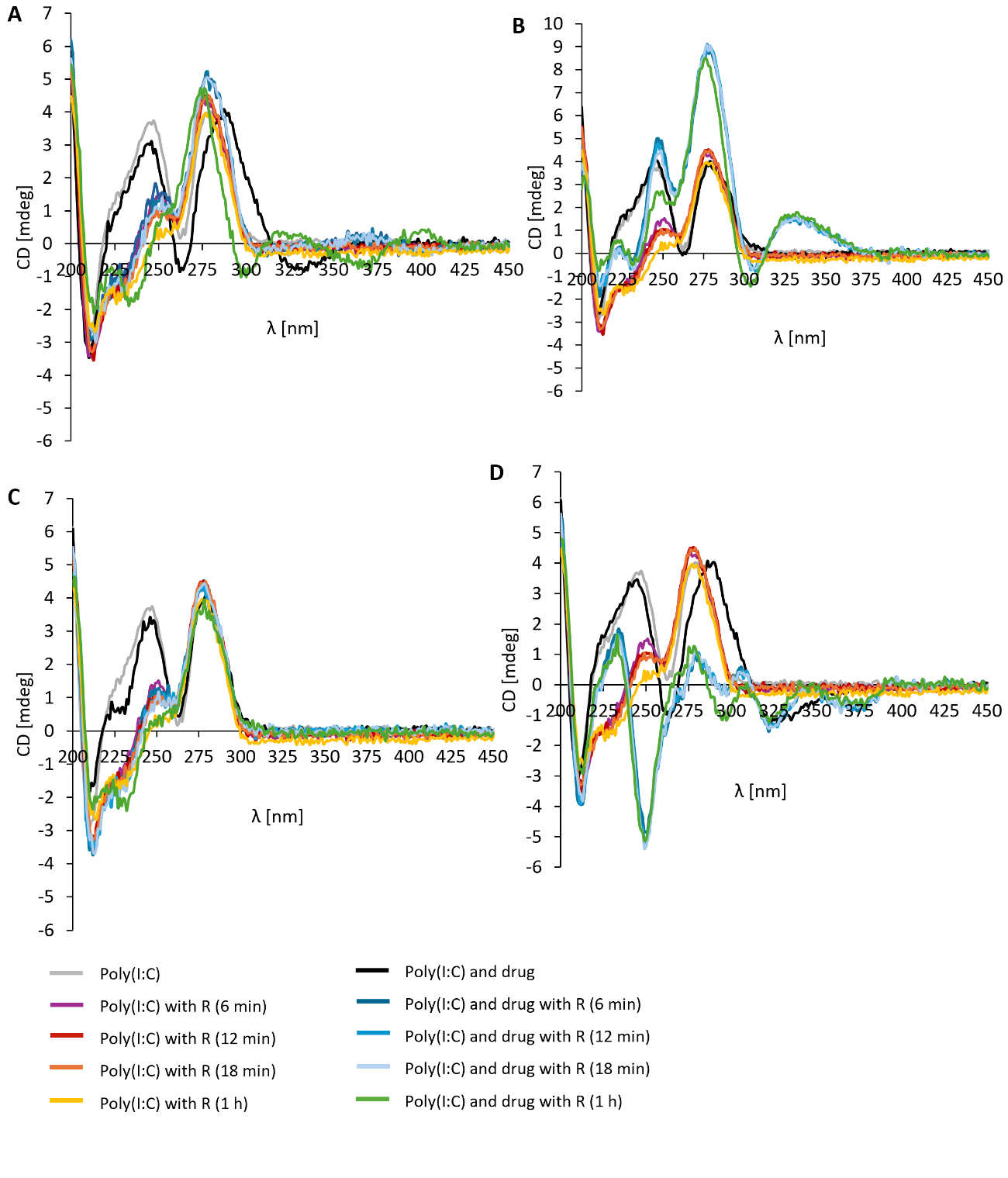


Figure S11: Effect of RNase on CD spectra of Poly(I:C) Na⁺ γ alone and with **(A)** doxycycline, **(B)** minocycline, **(C)** doxorubicin, **(D)** tetracycline in PBS.


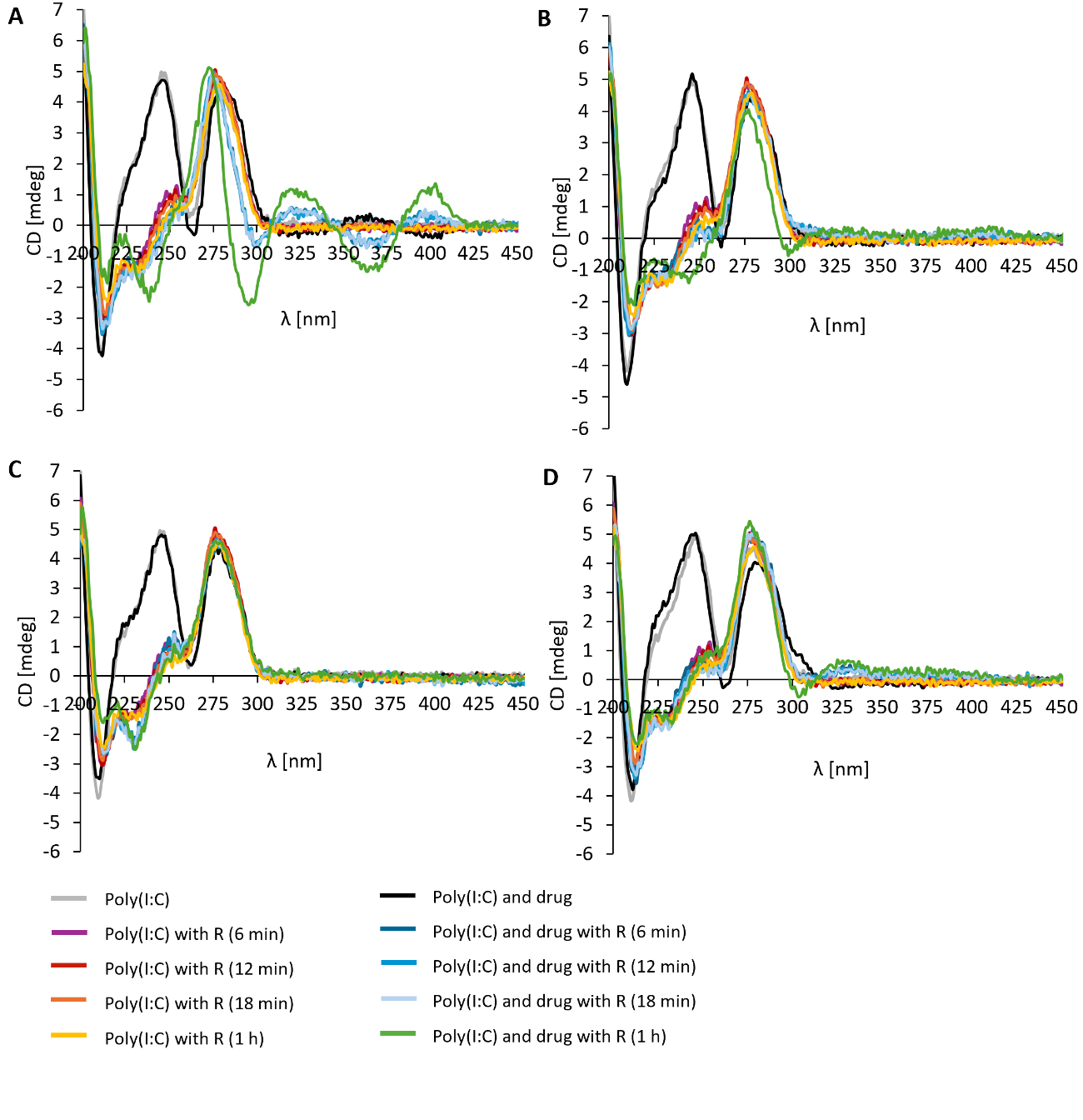


Figure S12: Effect of RNase on CD spectra of Poly(I:C) Na⁺ alone and with **(A)** doxycycline, **(B)** minocycline, **(C)** doxorubicin, **(D)** tetracycline in the presence of 10 mM MgSO_4_ in PBS.


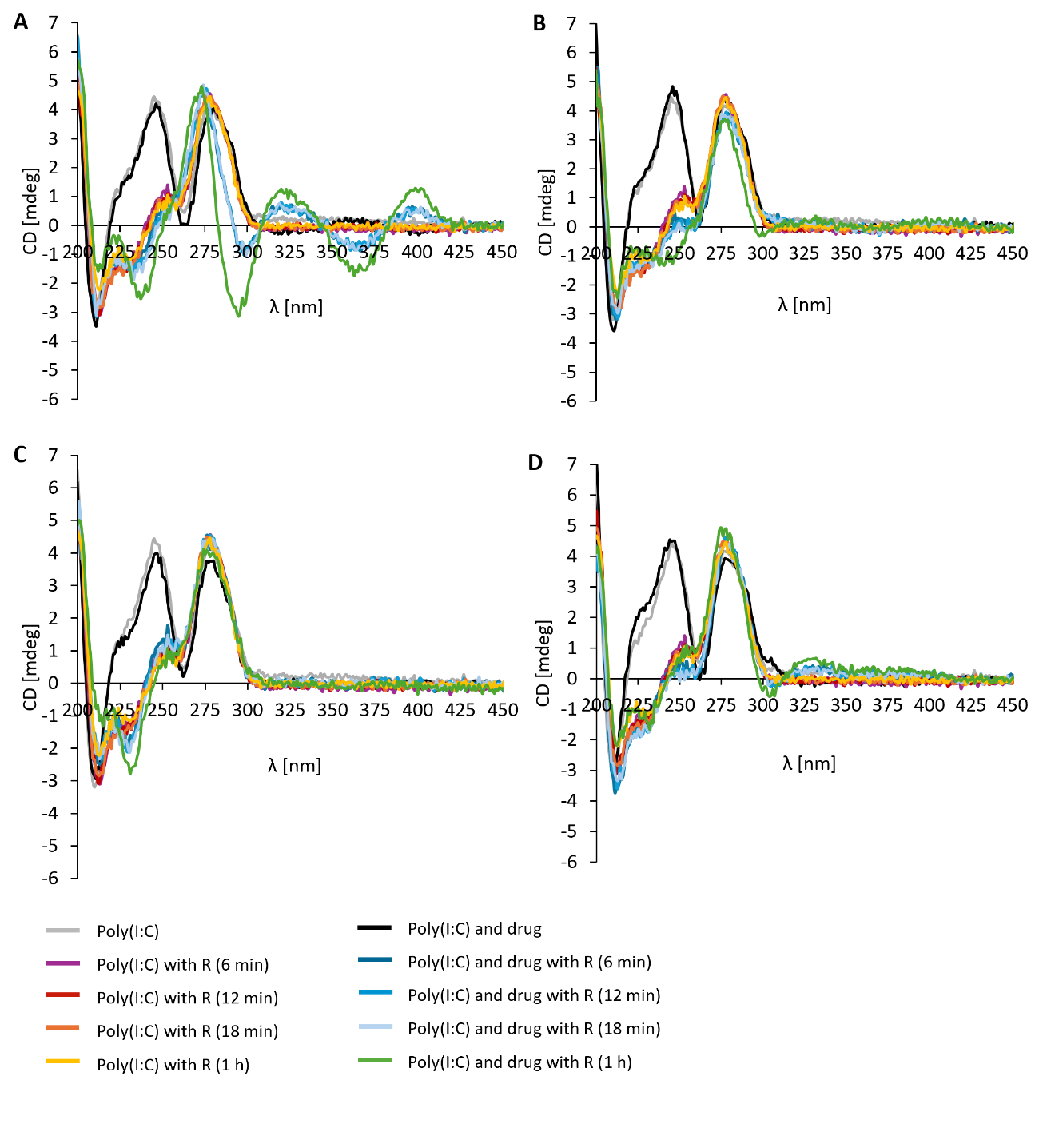


Figure S13: Effect of RNase on CD spectra of Poly(I:C) Na⁺ γ alone and with **(A)** doxycycline, **(B)** minocycline, **(C)** doxorubicin, **(D)** tetracycline in the presence of 10 mM MgSO_4_ in PBS.


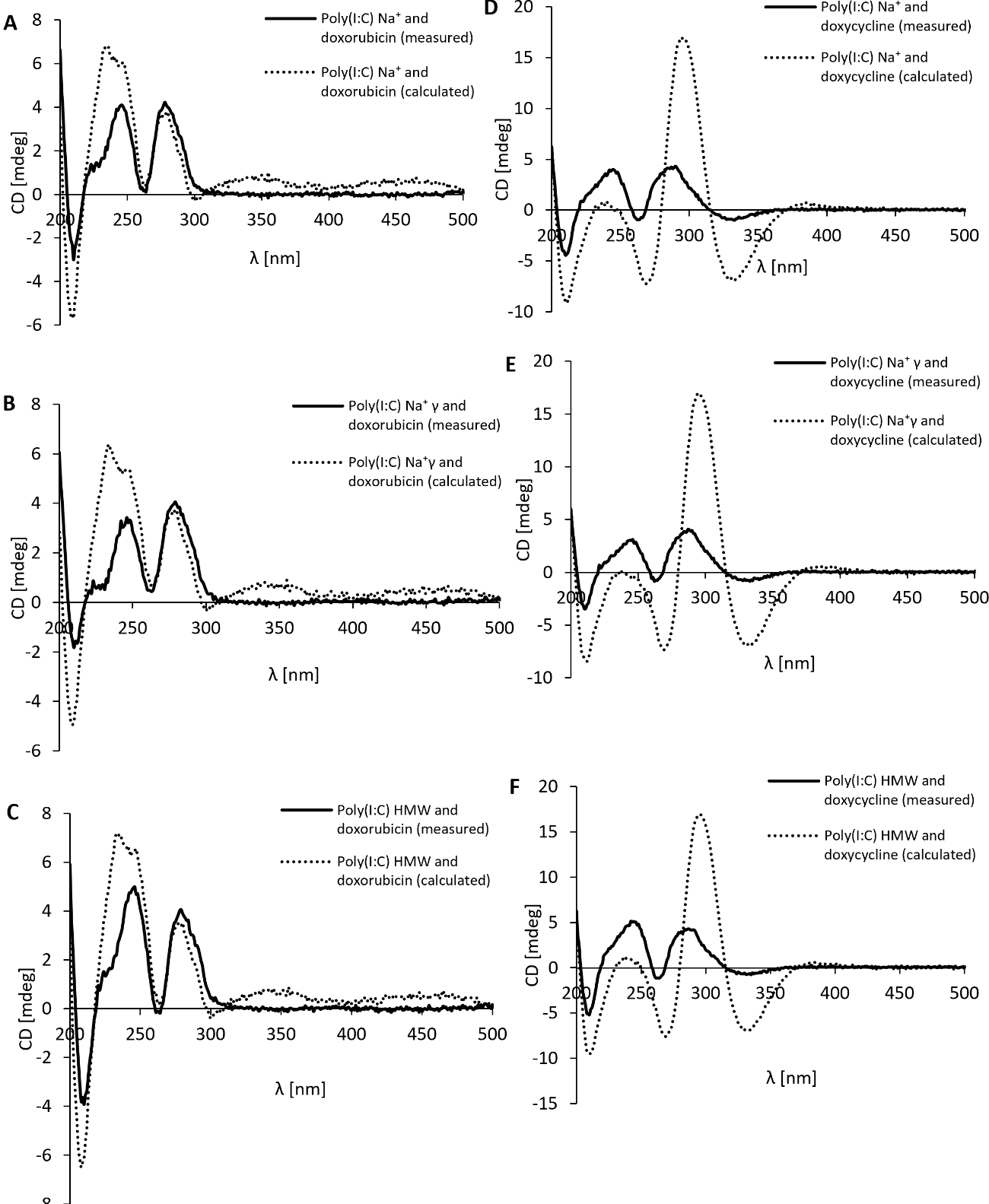


Figure S14: Qualitative binding interactions of doxorubicin with **(A)** Poly(I:C) Na^+^, **(B)** Poly(I:C) Na^+^ γ, **(C)** Poly(I:C) HMW and doxycycline with **(D)** Poly(I:C) Na^+^, **(E)** Poly(I:C) Na^+^ γ, **(F)** Poly(I:C) HMW.


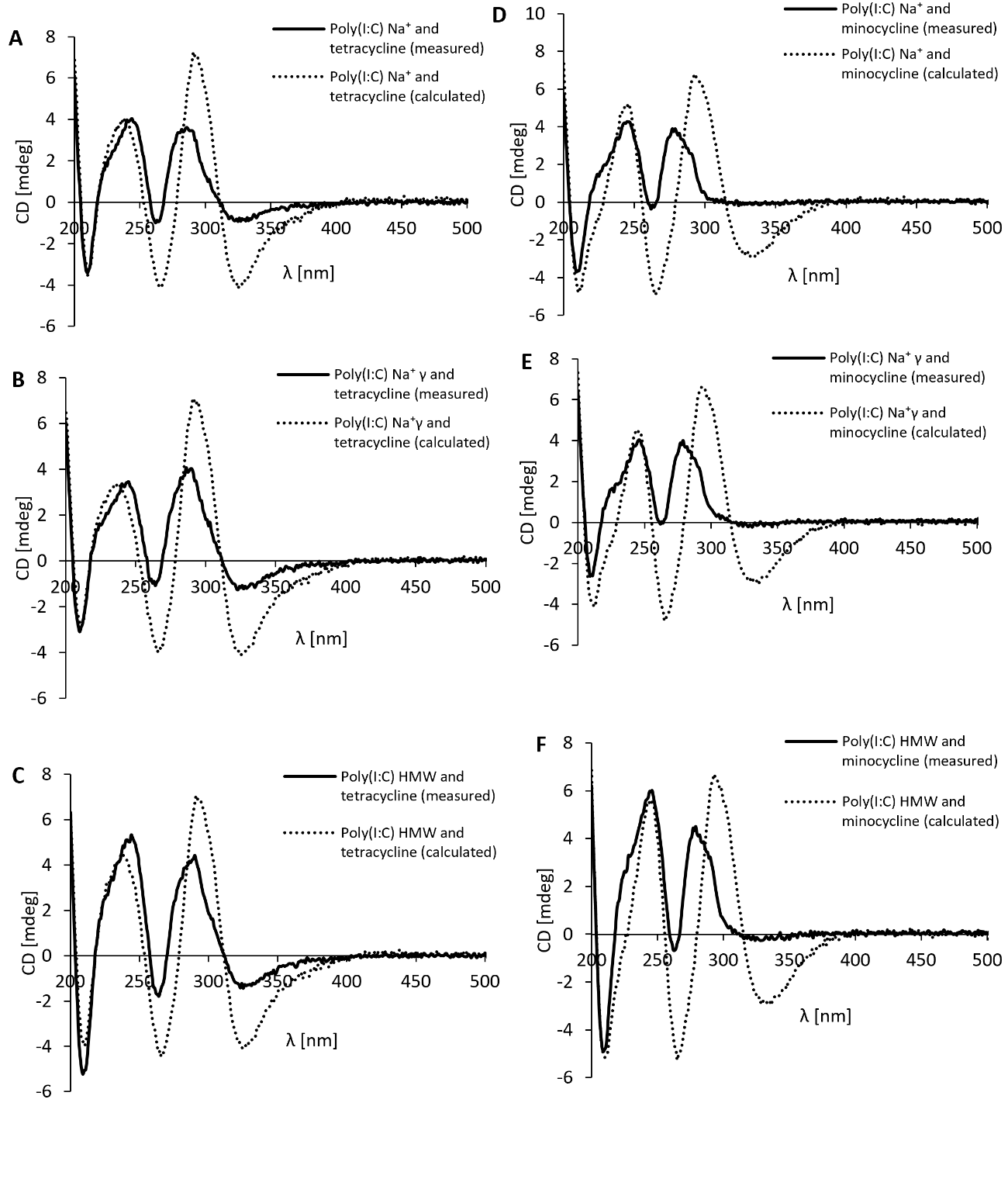


Figure S15: Qualitative binding interactions of tetracycline with **(A)** Poly(I:C) Na^+^, **(B)** Poly(I:C) Na^+^ γ, **(C)** Poly(I:C) HMW and minocycline with **(D)** Poly(I:C) Na^+^, **(E)** Poly(I:C) Na^+^ γ, **(F)** Poly(I:C) HMW.


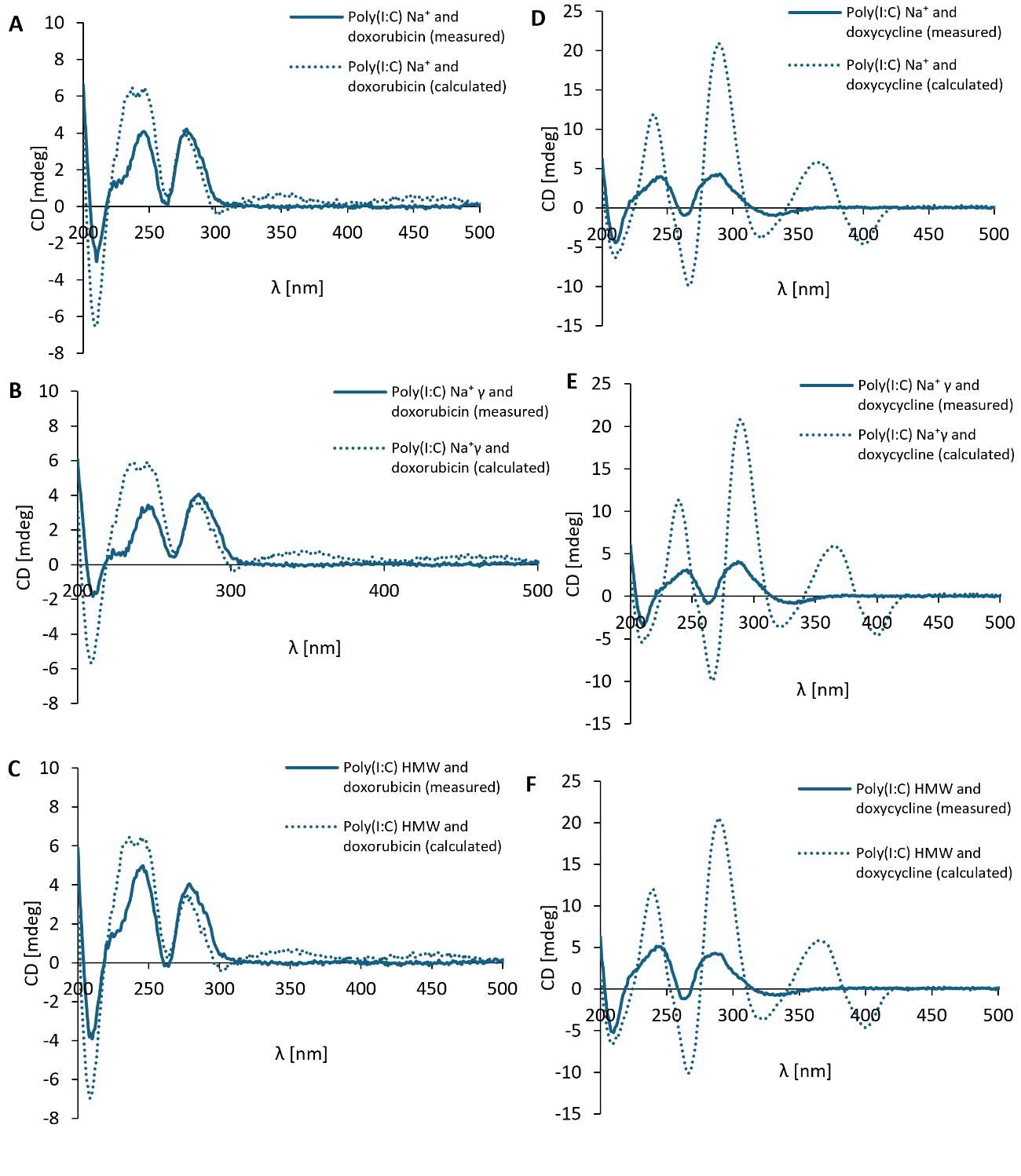


Figure S16: Qualitative binding interactions of doxorubicin with **(A)** Poly(I:C) Na^+^, **(B)** Poly(I:C) Na^+^ γ, **(C)** Poly(I:C) HMW and doxycycline with **(D)** Poly(I:C) Na^+^, **(E)** Poly(I:C) Na^+^ γ, **(F)** Poly(I:C) HMW in the presence of 10 mM MgSO_4_.


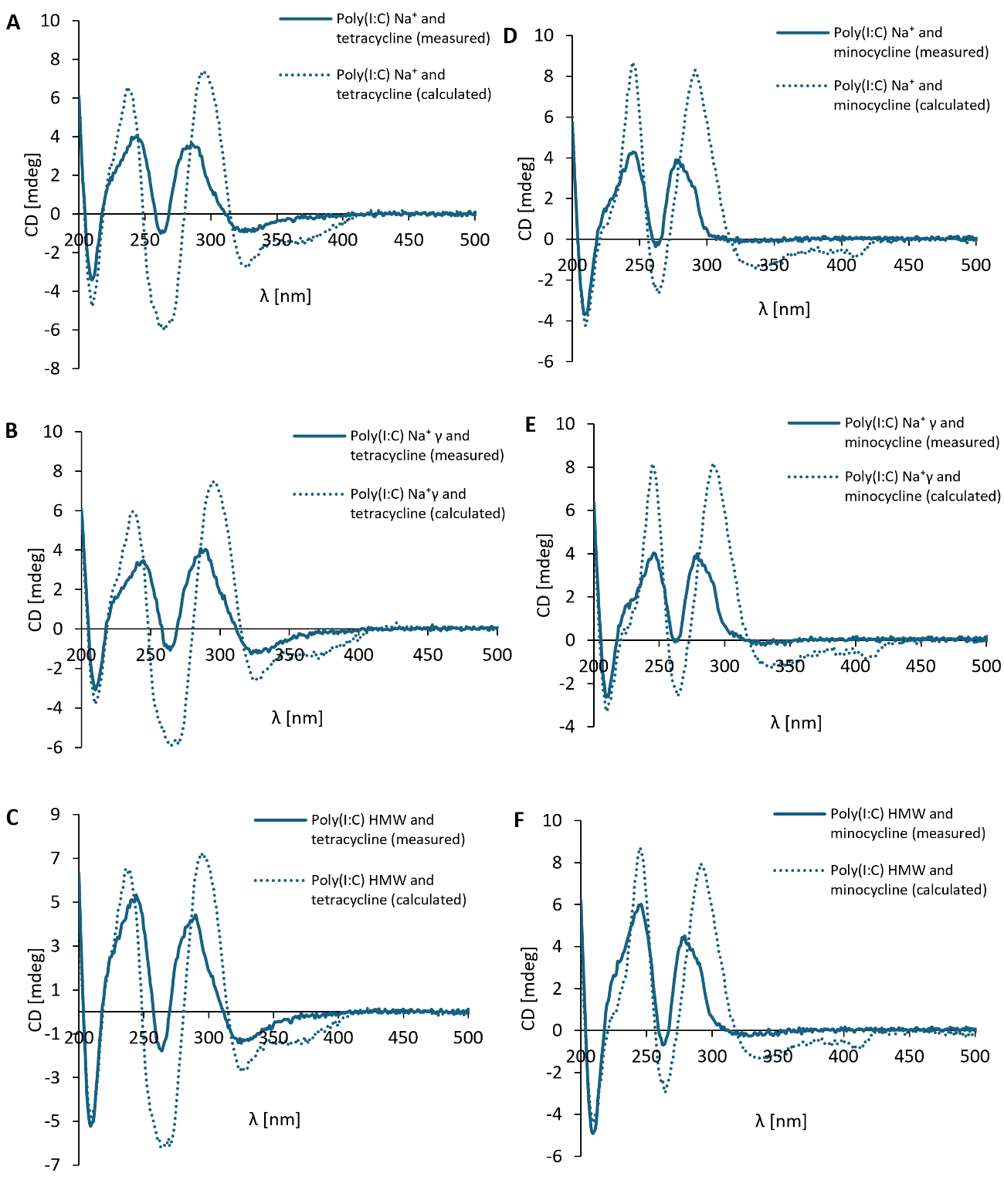


Figure S17: Qualitative binding interactions of tetracycline with **(A)** Poly(I:C) Na^+^, **(B)** Poly(I:C) Na^+^ γ, **(C)** Poly(I:C) HMW and minocycline with **(D)** Poly(I:C) Na^+^, **(E)** Poly(I:C) Na^+^ γ, **(F)** Poly(I:C) HMW in the presence of 10 mM MgSO_4_


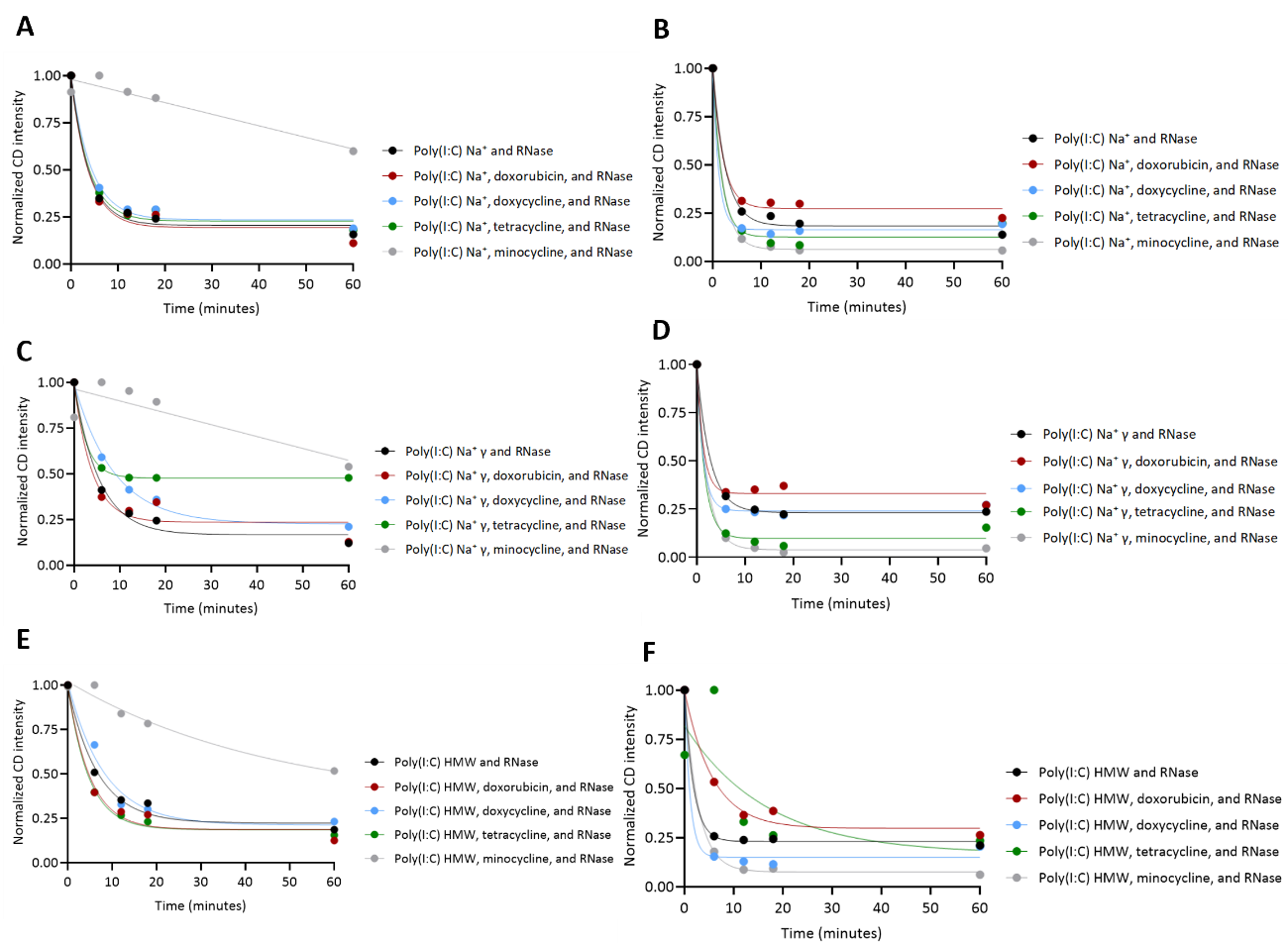


Figure S18: Normalized CD intensity of **(A)** Poly(I:C) Na⁺ and drugs, **(C)** Poly(I:C) Na⁺ γ and drugs, **(E)** Poly(I:C) HMW and drugs at absorbance maxima (around 250 nm) for each time point. Normalized CD intensity of **(B)** of Poly(I:C) Na⁺ and drugs, **(D)** Poly(I:C) Na⁺ γ and drugs, **(F)** Poly(I:C) HMW and drugs in the presence of 10 mM MgSO_4_ at absorbance maxima (around 250 nm) for each time point.

Table S1: The rate constant (K) and half-life (t_1/2_) of Poly(I:C) alone and in combination with chemotherapeutic drugs in the absence and presence of 10 mM MgSO_4._

| **With 0 mM MgSO_4_** | | | | | | | | | | | | | | |
| --- | --- | --- | --- | --- | --- | --- | --- | --- | --- | --- | --- | --- | --- | --- |
|  | **Poly(I:C) Na⁺** | | | | **Poly(I:C) Na⁺ γ** | | | | | **Poly(I:C) HMW** | | | | |
|  | **K** | | **Half-life** | | **K** | | **Half-life** | | **K** | | | **Half-life** | |  |
| alone | 0.2667 | | 2.599 | | 0.1787 | | 3.880 | | 0.1564 | | | 4.431 | |  |
| with doxorubicin | 0.3304 | | 2.098 | | 0.2500 | | 2.773 | | 0.1963 | | | 3.531 | |  |
| with doxycycline | 0.2550 | | 2.718 | | 0.1383 | | 5.011 | | 0.1226 | | | 5.652 | |  |
| with tetracycline | 0.2537 | | 2.732 | | 0.2573 | | 2.694 | | 0.2188 | | | 3.168 | |  |
| with minocycline | > 1 | | - | | > 1 | | - | | 0.02904 | | | 23.87 | |  |
| **With 10 mM MgSO4** | | | | | | | | | | | | | |  |
|  | | **Poly(I:C) Na⁺** | | | | **Poly(I:C) Na⁺ γ** | | | | | **Poly(I:C) HMW** | | |  |
|  |  | **K** | | **Half-life** | | **K** | | **Half-life** | | | **K** | | **Half-life** |  |
| alone | | 0.4054 | | 1.710 | | 0.3830 | | 1.810 | | | 0.4154 | | 1.669 |  |
| with doxorubicin | | 0.4897 | | 1.415 | | 0.3878 | | 1.787 | | | 0.1692 | | 4.096 |  |
| with doxycycline | | 0.5964 | | 1.162 | | 0.5208 | | 1.331 | | | 0.7243 | | 0.9570 |  |
| with tetracycline | | 0.5332 | | 1.300 | | 0.9817 | | 0.7060 | | | 0.06118 | | 11.33 |  |
| with minocycline | | 0.2628 | | 2.637 | | 0.6593 | | 1.051 | | | 0.2568 | | 2.699 |  |

## **Microscale Thermophoresis of Chemotherapeutics to a Human Nuclease and Poly(I:C) Molecule**


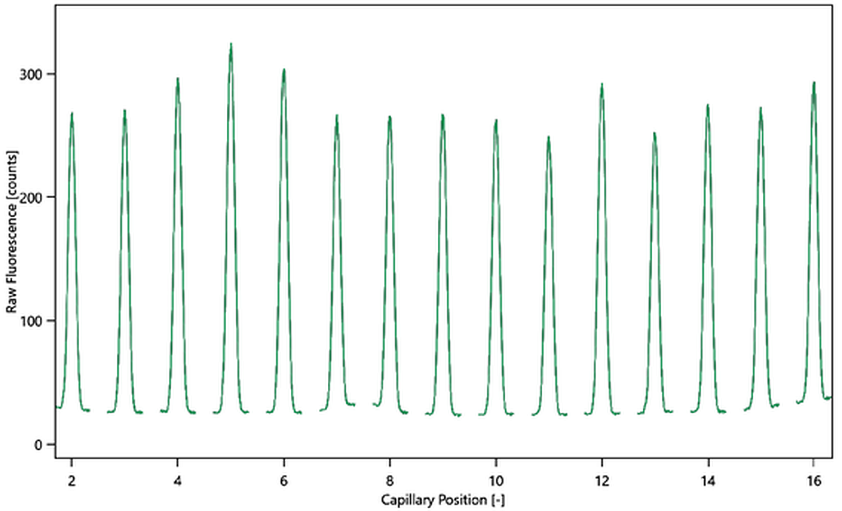


Figure S19: Capillary scan of MST measurement.


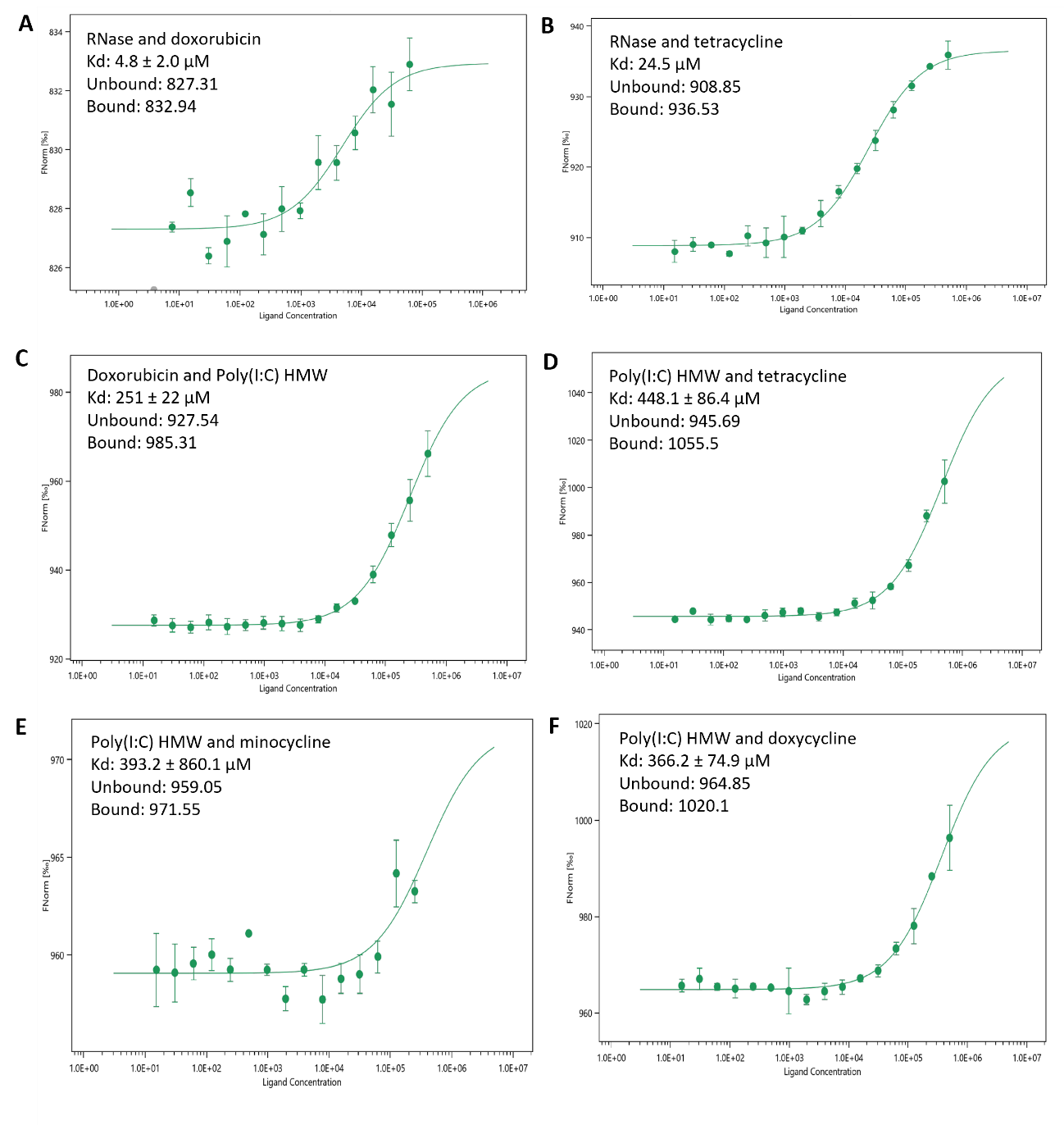


Figure S20: Binding of **(A)** doxorubicin, **(B)** tetracycline to a human RNase, and Kd values determined from the MST data. Binding of **(C)** doxorubicin, **(D)** tetracycline, **(E)** minocycline, and **(F)** doxycycline to Poly(I:C) HMW fluorescein, and Kd values determined from the MST data.

## **Cytotoxicity Assay of Minocycline and Doxycycline**


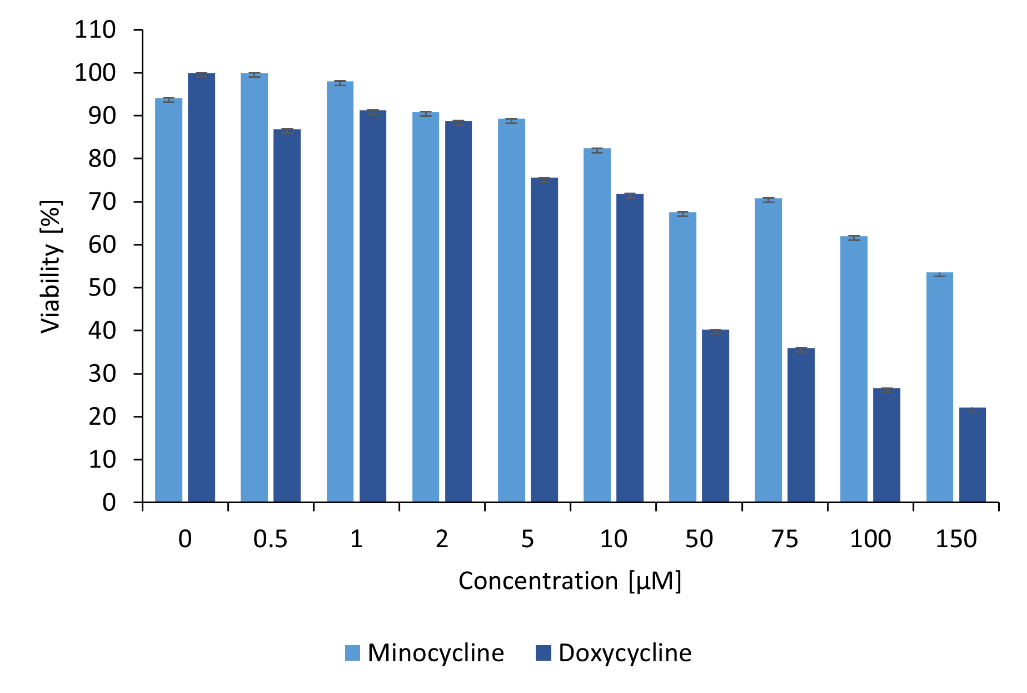


Figure S21: Cytotoxicity assay of minocycline and doxycycline on HEK dual hTLR3 cell line after 48 hours. Data are presented as mean ± SEM.

## **Dual NF-κB and IRF Assay**


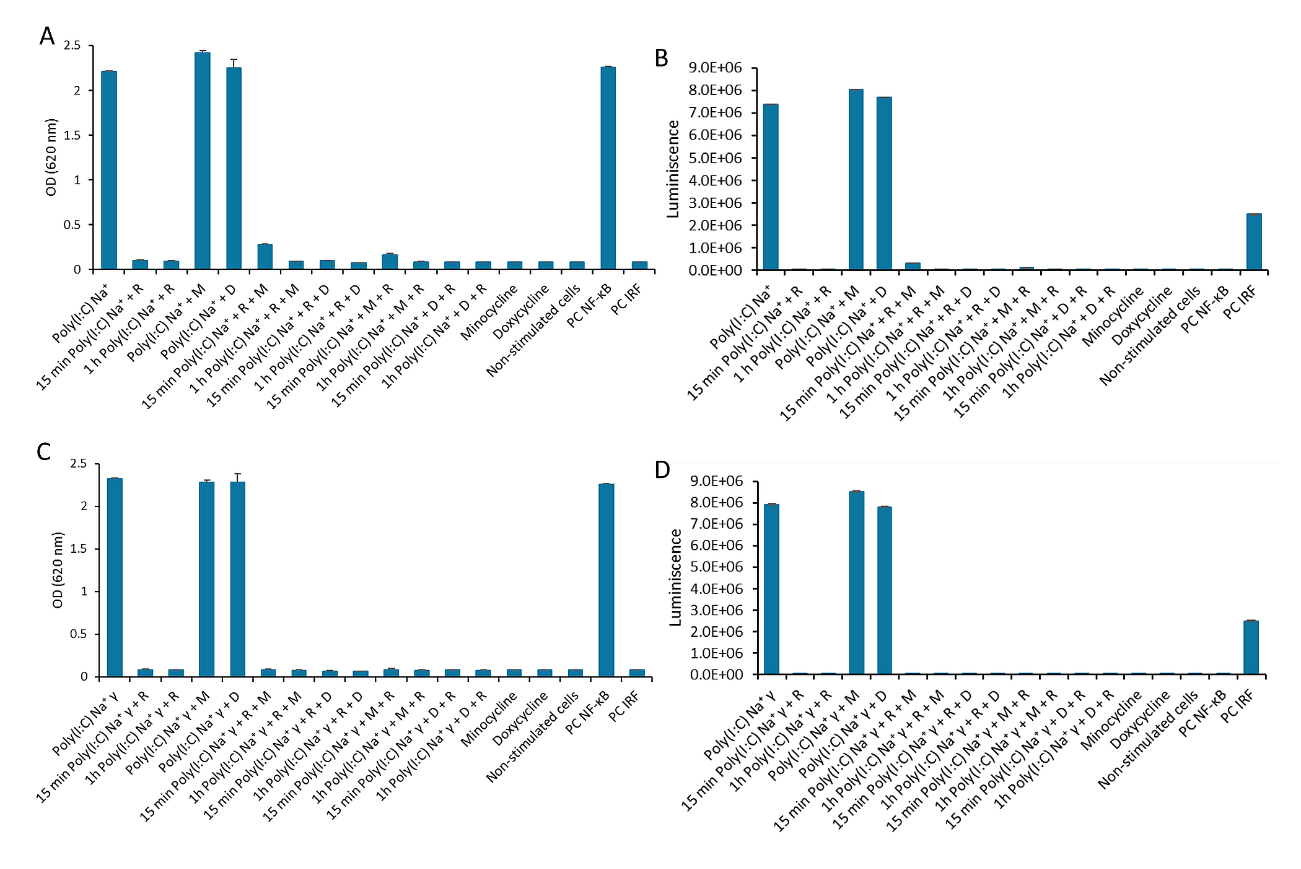


Figure S22: **(A)** NF-κB response of HEK dual hTLR3 cells to treatment with Poly(I:C) Na⁺+minocycline/doxycycline+RNase and Poly(I:C) Na⁺+RNase+minocycline/doxycycline. **(B)** Activation of IRF pathway upon Poly(I:C) Na⁺+minocycline/doxycycline+RNase and Poly(I:C) Na⁺+RNase+minocycline/doxycycline. **(C)** NF-κB response of HEK dual hTLR3 cells to treatment with Poly(I:C) Na⁺ γ+minocycline/doxycycline+RNase and Poly(I:C) Na⁺ γ+RNase+minocycline/doxycycline. **(D)** Activation of IRF pathway upon Poly(I:C) Na⁺ γ+minocycline/doxycycline+RNase and Poly(I:C) Na⁺ γ+RNase+minocycline/doxycycline. Data are presented as mean ± SEM.

# **MATERIALS AND METHODS**

## **Materials**

Chemicals and solvents used in this study were purchased from commercial suppliers such as Merck, TCI Chemicals, ThermoScientific, InvivoGen, InvitroGen, and VWR. Materials included doxorubicin hydrochloride (TCI Chemicals, purity >95%, cat. no. D4193, lot: RQAYJ), doxycycline hyclate (ThermoScientific, purity >95%, cat. no. J60579.14, lot: T26H014), tetracycline hydrochloride (Merck, 200 quality level, cat. no. T7660, lot: 0000181023), minocycline hydrochloride (Thermo Scientific Chemicals, purity 98.7 %, cat. no. J66429-ME, lot: X11D026), sodium salt of Poly(I:C) (Merck, 300 quality level, cat. no. P1530, source: 0000122152, batch: 0000125513), sodium gamma irradiated salt of Poly(I:C) (Merck, 300 quality level, cat. no. P0913, lot: 128M4040V), Poly(I:C) HMW (InvivoGen, cat. no. tltl-pic-5, batch: 5935-44-03, endotoxin-free 0.9% NaCl water included), Poly(I:C) HMW fluorescein (InvivoGen, cat. no. tlrl-picf, lot: 5939-45-02), His-Tag labeling kit RED-tris-NTA 2nd generation (NanoTemper Technologi), MgSO_4_ (Thermo Scientific Chemicals, purity 97%, cat. no. 413480050), dimethyl sulfoxide (DMSO) (VWR, purity ≥99.5% cat. no. 23500.297), 5 M NaCl in H_2_O (Merck, cat. no. S5150), phosphate-buffered saline (PBS) pH 7.4 (Merck, cat. no. 806552), nuclease-free deionized water (InvitroGen, cat. no. 10429224), Ambion RNase III (InvitroGen, cat. no. AM2290, lot: 3053867, 01305955), and recombinant ribonuclease III with N-terminal His-Tag (CLOUD-CLONE CORP., cat. no. RPD163Hu01, lot: P20240822641). For dual NF-κB and IRF assay and MTT assay, Penicillin-Streptomycin solution 100X (Biosera, cat. no. XC-A4122/100), fetal bovine serum (Biosera, cat. no. FB-1001/500), heat-inactivated fetal bovine serum (Biosera, cat. no. FB-1001H/500), Dulbecco's modified eagle medium (Sigma-Aldrich, cat. no. D6429-500ML), tissue culture water (Biosera, cat. no. T1707/500), human HEK-Dual hTLR3 Cells (InvivoGen, cat. no. hkd-htlr3, lot: V21-4501, blasticidin (10 mg/ml), hygromycin B gold (100 mg/ml), normocin (50 mg/ml), zeocin (100 mg/ml), QB reagent, QB buffer, QUANTI-Luc 4 reagent included), and MTT (3-(4,5-Dimethylthiazol-2-yl)-2,5-Diphenyltetrazolium Bromide)(Glentham Life Sciences, cat. no. GC4568) were used. Table S2 summarizes the size, solubility, or average size of commercially available Poly(I:C) molecules.

Table S2: Available information about commercial Poly(I:C) molecules.

|  | **Poly(I:C) Na^+^**  (Merck) | **Poly(I:C) Na^+^ γ**  (Merck) | **Poly(I:C) HMW**  (InvivoGen) |
| --- | --- | --- | --- |
| **Solubility/solvent** | 9.80 - 10.20 mg/mL in PBS, heated at 50 °C | 9.80 - 10.20 mg/mL in PBS, heated at 50 °C | 1 mg/mL in 0.9% NaCl,  heated at 65-70 °C |
| **Purity** | ≥ 99.00 % | ≥ 99.00 % | Confirmed absence of lipoproteins and endotoxins |
| **Average size** | Double-stranded homopolymer  Size not specified | Double-stranded homopolymer  Size not specified | Long double-stranded homopolymer  Size 1.5 - 8 kb |
| **Other** |  | Gamma-irradiation min. 2.5 Mrad (25 KGy) | - |

## **Stability of Selected Poly(I:C) Molecules**

UV-Vis spectroscopy was used to determine the stability of Poly(I:C) molecules over time and after repeated freeze-thaw cycles. Selected dsRNA analogs were sodium salt of Poly(I:C) [Poly(I:C) Na⁺], sodium gamma irradiated salt of Poly(I:C) [Poly(I:C) Na⁺ γ], and high molecular weight Poly(I:C) [Poly(I:C) HMW]. Stock solutions of all dsRNA analogs (1 mg/mL) were prepared according to manufacturers in 0.9% NaCl and heated up to 50 °C [for Poly(I:C) Na⁺ and Poly(I:C) Na⁺ γ] and 65 °C [for Poly(I:C) HMW] for 10 minutes. Cooling of samples to achieve proper reannealing of strands followed. All stock solutions were subsequently diluted in 0.9% NaCl (pH 5.8) to a concentration of 100 μg/mL. UV-Vis spectra were measured using Shimadzu UV-2401PC at 190-500 nm in a quartz cuvette with a lid and a 1 cm light pathway (Hellma Analytics, cat. no. 117200F-10-40) over one month and storage at 4 °C. Additionally, the effect of repeated freezing and thawing cycles of samples [up to 50 °C for Poly(I:C) Na⁺ and Poly(I:C) Na⁺ γ, and 65 °C for Poly(I:C) HMW] diluted in 0.9% NaCl over one month was studied. Stock solutions of Poly(I:C)s (1 mg/mL in 0.9% NaCl) were also diluted in PBS to the concentration of 100 μg/mL to better mimic the physiological conditions and measured at 190-500 nm in a quartz cuvette with a lid and a 1 cm light pathway stored at 4 °C for one week. Data spacing of 1 nm and a very slow scan rate mode were applied. UV-Vis spectra and absorbance maxima were analyzed in Microsoft Excel (version 2402).

## **Conditional Binding Constant and Stoichiometry between Chemotherapeutics and Poly(I:C) Molecules**

Fluorescence spectroscopy was selected for determining conditional binding constants and stoichiometry between each Poly(I:C) molecule and anthracycline or tetracycline chemotherapeutic in the presence and absence of 10 mM MgSO_4_ in PBS. Stock solutions of Poly(I:C)s (1 mg/mL) in 0.9% NaCl and chemotherapeutics (500 μg/mL) in DMSO were used. The concentration of each chemotherapeutic agent was held constant at 5×10^-6^ M (DMSO < 1 %). The molar concentration of Poly(I:C) was calculated from the molecular weight of Poly(I:C) base pair (671.4 g/mol) and varied in the range of 1.2×10^-6^ to 1.75×10^-4^ M. As solvents, in general, can tremendously affect the possible drug-drug or drug-ion interactions, titrations were conducted in an environment with the same DMSO:PBS ratio. Excitation maxima of each chemotherapeutic were selected based on the UV-Vis absorption spectra and were the following: 480 nm for doxorubicin hydrochloride, 353 nm for doxycycline hyclate, 364 nm for tetracycline hydrochloride, and 348 nm for minocycline hydrochloride (data not shown). The fluorescence emission spectra were measured using a Shimadzu RF-6000 spectrofluorimeter in a quartz cuvette with a lid and a 1 cm light pathway (Hellma Analytics, cat. no.117200F-10-40) at laboratory temperature. Data spacing of 1 nm and a scan rate of 60 nm/min were applied.

Conditional binding constants (log K) were calculated with Letagrop SPEFO 2005 software and derived from the following equation: K = c_complex_/(c_polymer_*^n^*xc_ligand_^m^), as described in detail elsewhere ^1^, where *c* represents the concentrations of the complex, Poly(I:C) and chemotherapeutic drug, *n,* and *m* represent the number of Poly(I:C) and chemotherapeutic molecules in the complex, respectively. As Poly(I:C) chains have various lengths, concentrations of Poly(I:C) molecules were expressed using concentrations of each repeating base pair.

## **UV-Vis Melting of Poly(I:C)-chemotherapeutic Complexes**

UV-Vis spectroscopy was used to obtain melting curves of Poly(I:C), chemotherapy, and Poly(I:C)-chemotherapy samples in the absence and presence of 10 mM MgSO_4_. Stock solutions of Poly(I:C)s in 0.9% NaCl (1 mg/mL) and chemotherapeutic agents (500 μg/mL) were diluted in PBS (pH 7.4) to a final concentration of 100 μg/mL and 5×10^-5^ M, respectively. In the case of Poly(I:C)-chemotherapy measurements, Poly(I:C)s, and chemotherapeutics were diluted in PBS to a final concentration of 5×10^-5^ M (1:1). The concentration of DMSO was less than 6%. Samples (1,400 µl) were then placed in a quartz cuvette (Starna Scientific, Spectrosil Far UV Quartz cuvette, type: 9B/Q/10) with a path length of 1 cm and analyzed by SPECORD 50 PLUS, Anakytik Jena. Absorbance spectra of Poly(I:C) molecules and chemotherapeutics alone were measured with a step of 1 nm and a scan rate of 1 nm/sec every 5 °C with a precision of 0.1 °C in the 20-90 °C temperature range. Absorption spectra of chemotherapeutics were compared at absorbance maxima of each Poly(I:C) to correctly select the wavelength for Poly(I:C)-chemotherapy measurements and were following: 267 nm for Poly(I:C) Na^+^, 266 nm for Poly(I:C) Na^+^ γ, and 264 nm for Poly(I:C) HMW in the absence and presence of 10 mM MgSO_4_. Absorbance spectra of Poly(I:C)-chemotherapeutics were measured simultaneously by 1 °C from 20 to 90 °C with a scan rate of 1 nm/min and precision of 0.1 °C. Melting temperatures were determined numerically from the first derivative plot in Microsoft Excel (version 2402).

## **In Silico Docking of Chemotherapeutics (and of Poly(I:C)) to a Human Nuclease**

For the docking calculations, the three-dimensional (3D) crystal structure of the C-Terminal RNase III Domain of Human Dicer (RIIID) was downloaded from the Protein Data Bank (PDB id 2EB1). Additionally, we retrieved the coordinates of a high-resolution 3D structure of an RIIID dimer in a complex with double-stranded RNA (PDB id 2NUG). Using CCP4MG (version 2.10.11), we superposed the structure of 2NUG onto the 2EB1 dimer. This superposition correctly positioned the double-stranded RNA relative to the 2EB1 dimer. The RNA coordinates from the superposed structure were extracted and inserted into the coordinate file of the 2EB1 dimer to provide a model of double-stranded RNA bound to the RIIID dimer. This model was then subjected to energy minimization using the YASARA web server. For modeling the double-stranded poly(I:C), the coordinates of the RNA analog were obtained from the PDB 7WV5. Using Coot (version 0.8.9), the Poly(I:C) was shortened to contain 18 base pairs with two sticky ends on opposite strands. Finally, we used the “align” function in PyMOL (version 2.5.2) to superpose the modified double-stranded Poly(I:C) onto the RNA in the complex. The 3D structures of ligands (doxorubicin hydrochloride, tetracycline hydrochloride, minocycline hydrochloride, and doxycycline hyclate) were obtained from the PubChem open chemistry database and saved in the .pdb format using UCSF Chimera version 1.15.

Prior to the docking procedure, the 3D models were manually modified by removing crystallographic waters, ligands, and other unnecessary components (double conformations) using UCSF Chimera. Molecular docking calculations between the RNAse III D receptor and the ligands were performed with the AutoDock Vina suite. The calculations were carried out using the parameters recommended in the user manual. AutoDockTools (ADT version 1.5.7) was employed to find and determine the center and size of the grid box for the docking calculations. The predicted binding affinities (kcal/mol) were calculated with Auto-Dock Vina. To generate overall views of the docking outcomes, UCSF Chimera was used, while BIOVIA Discovery Studio Visualizer (version 21.1.0.20298) was utilized to create two-dimensional diagrams and illustrate the interactions of the ligands with amino acids. PyMOL was employed to visualize the positioning of the ligand on the receptor surface.

## **Thermal Stability of RNase III**

The thermal stability of Ambion RNase III was determined using a differential scanning fluorimeter (Prometheus NT.48, Nano Temper) accessed through CIISB at the Centre of Molecular Structure, BIOCEV. Ambion RNase III sample was diluted in PBS (1:1). Tryptophan fluorescence around 330 and 350 nm was detected during the sample warming from 20 to 85 °C. The excitation power of 60% and 1.0 °C/min temperature slope were applied.

## **Circular Dichroism of Poly(I:C) Molecules with Chemotherapeutics**

The secondary structure of selected Poly(I:C)s and changes in CD spectra of Poly(I:C)s in combination with chemotherapeutics were studied by circular dichroism spectrometry in the absence and presence of 10 mM MgSO_4_. Stock solutions of Poly(I:C)s in 0.9% NaCl (1 mg/mL) and chemotherapeutics in deionized water (500 μg/mL) were diluted in PBS to a concentration 1×10^-4^ M (1:1). 160 µL of the sample was placed in a quartz cuvette with a path length of 1 mm (Hellma Analytics, cat. no. HL110-1-40). Samples were measured in the range of 200-650 nm at 37 °C with a 1 nm step size, 1nm bandwidth, and 1s time per point using Chirascan Plus, Applied Photophysics. The possible protection of chemotherapeutics against Poly(I:C)s’ degradation was also determined. Ambion RNase III was applied according to the manufacturer’s protocol. CD spectra upon the addition of RNase III were immediately measured in the range of 200-650 nm at 37 °C, three times every 6 minutes, and after 1 hour with a 1 nm step size, 1 nm bandwidth, and 0.5s time per point. Data was analyzed using Microsoft Excel, version 2411. Furthermore, GraphPad Prism 8.0.1 was used for the analysis of the stability degree and calculation of the rate of degradation via non-linear regression, one-phase exponential decay. Absorbance maxima (around 250 nm) for each time point were selected for the analysis.

## **Microscale Thermophoresis of Chemotherapeutics to a Human Nuclease and Poly(I:C) Molecule**

Microscale thermophoresis was performed to experimentally verify in silico docking data. Dissociation constants of selected chemotherapeutics to Poly(I:C) HMW fluorescein and a human his-tagged RNase were measured in PBS-T buffer (pH 7.4). His-tagged RNase (10 μg) was reconstituted in PBS to a concentration of 0.1 mg/mL according to the manufacturer and adjusted to a concentration of 200 nM. The His-Tag labeling kit was used according to the manufacturer’s protocol for fluorescent labeling of the His-tagged RNase. Poly(I:C) HMW fluorescein required no further labeling, and concentration was adjusted to 200 nM. Sixteen-step dilution was followed and performed by adding 10 μL of PBS-T into PCR tubes 2-16. 20 μL of a ligand (1 mM for doxycycline, minocycline, and tetracycline, and 0.25 mM for doxorubicin) was then placed into tube 1. Half the volume from tube 1 was transferred into well 2 and mixed thoroughly. The procedure was repeated until tube 16 where the extra 10 µL was discarded from the tube. Sixteen samples were subsequently loaded into NT.115 standard capillaries. The first capillary contained the highest concentration of the ligand (500 nM for doxycycline, minocycline, tetracycline, and 125 nM for doxorubicin), and the sixteenth one contained the lowest concentration (15 nM for doxycycline, minocycline, tetracycline, and 4 nM for doxorubicin). Measurements were performed using a Monolith NT.115 device (Nano Temper Technologies GbmH), which we were granted access to through CIISB at the Centre of Molecular Structure, BIOCEV. Various excitation (EP) and MST powers were applied (for doxorubicin and RNase: EP 40%, MST 60%; for doxycycline and RNase: EP 40%, MST 40%; for minocycline and RNase: EP 50%, MST 60%; for tetracycline and RNase: EP 50%, MST 40%; for Poly(I:C) HMW and doxorubicin: EP 20%, MST 40%; for Poly(I:C) HMW and doxycycline: EP 30%, MST 40%; for Poly(I:C) HMW and minocycline: EP 30%, MST 40%; and Poly(I:C) HMW and tetracycline: EP 30%, MST 40%). The red laser was used for His-tagged RNase-chemotherapeutics measurements, and the green laser for the excitation of fluorescein-labeled Poly(I:C) HMW-chemotherapeutics samples. Due to the interference of fluorescence of doxorubicin hydrochloride and Poly(I:C) HMW fluorescein, Poly(I:C) HMW fluorescein was exchanged for non-labeled Poly(I:C) HMW. Data were analyzed via MO. Affinity Analysis version 2.2.4 software (Technologies GmbH; München, Deutschland).

## **Cytotoxicity Assay of Minocycline and Doxycycline**

A colorimetric MTT assay was selected to assess the cytotoxicity of minocycline hydrochloride and doxycycline hyclate in human HEK-Dual hTLR3 cells. The cells were cultured at 37 °C in 5% CO_2_ in DMEM culture medium supplemented with 10% heat-inactivated FBS, 1% Penicillin-Streptomycin, and selective antibiotics 10 μg/mL of Blasticidin, 100 μg/mL of Hygromycin B Gold, and 100 μg/mL of Zeocin. When cells reached 80% confluency, they were placed in 96-well plates in DMEM medium without the selective antibiotics at a density of 25,000 cells/well and incubated overnight at 37 °C in 5% CO_2_. After 24 hours, the cells were treated with increasing concentrations of minocycline and doxycycline in 200 μL of DMEM medium and incubated for an additional 48 hours. After two days, the medium was aspirated and exchanged for MTT tetrazolium dye [3-(4,5-dimethylthiazol-2-yl)-2,5-diphenyltetrazolium bromide] at a concentration of 5 mg/mL in fresh medium and left for 3 hours at 37 °C in 5% CO_2_. The tetrazolium dye was then removed, purple formazan crystals were dissolved in 100 μL of DMSO per well, and the absorbance was measured at a wavelength of 570 nm using a Spark microplate reader (accessed through CIISB at the Centre of Molecular Structure, BIOCEV). The experiment was performed in triplicates three times. The half-maximal inhibitory concentration (IC_50_) was calculated from dose-response curves using GraphPad Prism 8.0.1 (GraphPad Software, CA, USA). Figures were created using Microsoft Excel (version 2409). Data are presented as mean ± SEM.

## **Dual NF-κB and IRF Assay**

Human HEK-dual hTLR3 cells were cultured at 37 °C in 5% CO_2_ in DMEM culture medium supplemented with 10% heat-inactivated FBS, 1% Penicillin-Streptomycin, and selective antibiotics 10 μg/mL of Blasticidin, 100 μg/mL of Hygromycin B Gold, and 100 μg/mL of Zeocin. When cells reached 80% confluency, 20 μL of 1 μg/mL of Poly(I:C) Na⁺, Poly(I:C) Na⁺ γ, Poly(I:C) HMW at alone, 1 μg/mL of minocycline hydrochloride, and doxycycline hyclate alone or in combinations (1 μg/mL, 1:1), and after RNase treatment were added per well of a flat-bottom 96-well transparent plate. Two groups of RNase treatment were performed, including the prior incubation of Poly(I:C) with RNase for 15 minutes and 1 hour, with subsequent addition of doxycycline/minocycline, and addition of RNase to Poly(I:C)-chemotherapy samples. 10 ng/mL of TNF-α, an NF-κB positive control, and 1000 U/mL of IFN-β, an IRF positive control, and tissue culture water, a negative control, were included. Cells were placed to the treatments in 96-well plates in DMEM medium without the selective antibiotics at a density of 50,000 cells/well and incubated overnight at 37 °C in 5% CO_2_$.$ After 24 hours, the QUANTI-blue solution for the detection of the NF-κB response and the QUANTI-luc 4 reagent for the detection of the IRF response were prepared according to the manufacturer’s protocol. 20 µL of cell supernatant from each well was then transferred into a new flat-bottom 96-well transparent plate and a flat-bottom 96-well white plate for colorimetric and luminescence assay, respectively. 180 µL of QUANTI-blue solution was added to the cell supernatants in a transparent 96-well plate and incubated at 37 °C for one hour. SEAP levels were determined using a Spark microplate reader accessed through CIISB at 620-655 nm. For the detection of IRF response, 50 μL of the QUANTI-luc 4 reagent was added to cell supernatants in the flat-bottom 96-well white plate and determined using a Spark microplate reader with 50 ms integration time. Statistical analysis was conducted using GraphPad Prism 8.0.1 (GraphPad Software, CA, USA). The unpaired T-test was used to determine the significance of differences between the two groups. Data are presented as mean ± SEM. P-values ≤ were considered statistically significant (*≤ 0.5, **≤ 0.01, ***≤ 0.001, ****≤ 0.0001). Figures were created using Microsoft Excel (version 2409).

# **REFERENCES**

(1) Sillen, L. G. High-speed Computers as a supplement to Graphical Methods. *Acta Chem. Scand* **1962**, *16* (1), 159-172.
